# Paracrine signalling during ZEB1-mediated epithelial–mesenchymal transition augments local myofibroblast differentiation in lung fibrosis

**DOI:** 10.1038/s41418-018-0175-7

**Published:** 2018-07-26

**Authors:** Liudi Yao, Franco Conforti, Charlotte Hill, Joseph Bell, Leena Drawater, Juanjuan Li, Dian Liu, Hua Xiong, Aiman Alzetani, Serena J. Chee, Ben G. Marshall, Sophie V. Fletcher, David Hancock, Mark Coldwell, Xianglin Yuan, Christian H. Ottensmeier, Julian Downward, Jane E. Collins, Rob M. Ewing, Luca Richeldi, Paul Skipp, Mark G. Jones, Donna E. Davies, Yihua Wang

**Affiliations:** 1 Biological Sciences, Faculty of Natural and Environmental Sciences, University of Southampton, Southampton, SO17 1BJ UK; 2 Clinical and Experimental Sciences, Faculty of Medicine, University of Southampton, Southampton, SO16 6YD UK; 3 NIHR Southampton Biomedical Research Centre, University Hospital Southampton, Southampton, SO16 6YD UK; 4 Department of Oncology, Tongji Hospital, Tongji Medical College, Huazhong University of Science and Technology, Wuhan, 430030 China; 5 University Hospital Southampton, Southampton, SO16 6YD UK; 6 Cancer Sciences & NIHR and CRUK Experimental Cancer Sciences Unit, University of Southampton, Southampton, SO16 6YD UK; 7 Oncogene Biology, The Francis Crick Institute, London, NW1 1AT UK; 8 Unità Operativa Complessa di Pneumologia, Università Cattolica del Sacro Cuore, Fondazione Policlinico A. Gemelli, Rome, Italy; 9 Centre for Proteomic Research, Institute for Life Sciences University of Southampton, Southampton, SO17 1BJ UK; 10 Department of Thoracic Surgery, University Hospital Southampton, Southampton, SO16 6YD UK; 11 Institute for Life Sciences, University of Southampton, Southampton, SO17 1BJ UK

**Keywords:** Respiratory tract diseases, Predictive markers

## Abstract

The contribution of epithelial–mesenchymal transition (EMT) to human lung fibrogenesis is controversial. Here we provide evidence that ZEB1-mediated EMT in human alveolar epithelial type II (ATII) cells contributes to the development of lung fibrosis by paracrine signalling to underlying fibroblasts. Activation of EGFR–RAS–ERK signalling in ATII cells induced EMT via ZEB1. ATII cells had extremely low extracellular matrix gene expression even after induction of EMT, however conditioned media from ATII cells undergoing RAS-induced EMT augmented TGFβ-induced profibrogenic responses in lung fibroblasts. This epithelial–mesenchymal crosstalk was controlled by ZEB1 via the expression of tissue plasminogen activator (tPA). In human fibrotic lung tissue, nuclear ZEB1 expression was detected in alveolar epithelium adjacent to sites of extracellular matrix (ECM) deposition, suggesting that ZEB1-mediated paracrine signalling has the potential to contribute to early fibrotic changes in the lung interstitium. Targeting this novel ZEB1 regulatory axis may be a viable strategy for the treatment of pulmonary fibrosis.

## Introduction

Epithelial–mesenchymal transition (EMT), a dynamic and reversible biological process by which epithelial cells lose their cell polarity and down-regulate cadherin-mediated cell–cell adhesion to gain migratory properties, is involved in embryonic development, wound healing, fibrosis and cancer metastasis [1]. EMT is executed in response to pleiotropic signalling factors, including the transforming growth factor β (TGFβ) superfamily, Sonic Hedgehog (Shh), Wnt/β-catenin, fibroblast growth factor (FGF) and epidermal growth factor (EGF). These factors regulate the expression of specific transcription factors (TFs) called EMT-TFs (e.g. Snail, ZEB, Twist and others) that promote repression of epithelial features and induction of mesenchymal characteristics [2, 3]. Unlike EMT in cancer, which is detrimental, wound-healing-driven EMT induced in response to injury is beneficial, but exaggerated healing responses can lead to fibrosis or tissue scarring.

Fibrosis is a hallmark of many chronic degenerative disorders and is associated with reduced organ function and eventual organ failure. Fibrotic disease is on the increase; for example, idiopathic pulmonary fibrosis (IPF), the most common type of idiopathic interstitial pneumonia, occurs with similar frequency to that of stomach, brain and testicular cancer [4]. IPF is now generally regarded as a consequence of multiple interacting genetic and environmental risk factors, with repetitive local micro-injuries to ageing alveolar epithelium playing a central role [5]. These micro-injuries initiate the progressive accumulation of extracellular matrix (ECM) deposited by myofibroblasts. The origin of these myofibroblasts has been debated for many years, with EMT being considered as a potential source by driving the transformation of epithelial cells into ECM producing myofibroblasts [6–10]. However, lineage tracing in transgenic mice indicates that the contribution of those cells to the population of myofibroblasts is negligible [11–14].

In this study, we identify a novel regulatory axis involved in lung fibrosis whereby EMT contributes to the fibrotic process via paracrine activation of fibroblasts. We demonstrate that epidermal growth factor receptor (EGFR)-RAS-extracellular signal-regulated kinase (ERK) signalling induces the transcription factor ZEB1, which not only controls EMT but also regulates the production of locally-acting mediators. Specifically, we identified tissue plasminogen activator (tPA) as a downstream effector of ZEB1 transcriptional activity that contributes to paracrine signalling by enhancing TGFβ-induced profibrogenic responses in fibroblasts. Consistent with this, increased ZEB1 nuclear expression was detected in alveolar epithelium adjacent to sites of ECM deposition in IPF lung tissue. Thus, rather than contributing directly to the mesenchymal population, our data suggest that ZEB1-dependent EMT of ATII cells contributes to fibrosis via epithelial–fibroblast crosstalk. The occurrence of ZEB1 activation at sites of local ECM deposition in IPF lung tissue is consistent with the concept that ZEB1-regulated paracrine signalling contributes to the development of a profibrogenic microenvironment leading to interstitial lung fibrosis.

## Results

### Activation of EGFR signalling induces EMT in alveolar epithelial cells

To investigate IPF associated signalling pathways, we analysed differentially expressed genes in IPF and control lung tissue from a publicly available microarray dataset (GSE24206) [15]. Using a false discovery rate (FDR) corrected *P* value of 0.05, we identified 7668 genes to be differentially expressed out of a total of 54,675 probe sets. Gene network analysis using the Consensus Pathways Database [16] identified a number of pathways. Of these the EGFR–ERK pathway was the top-ranked pathway with 150 of 458 pathway candidates being significantly (*Q*-value < 0.05) overrepresented in the dataset (Supplementary Fig. S1a).

**Fig. 1:**
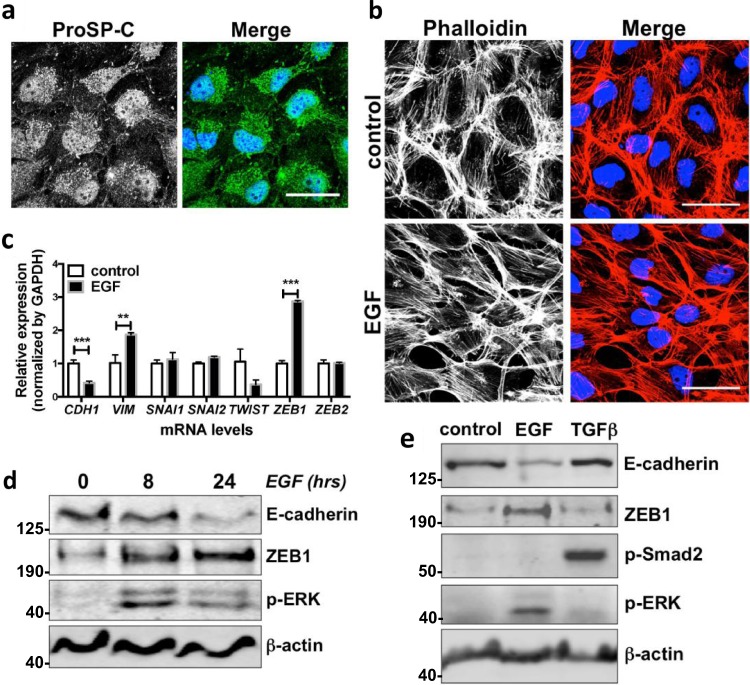
Activation of EGFR signalling induces EMT in alveolar epithelial cells. **a** Immunofluorescence staining of Pro-surfactant protein-C (Pro-SP-C) (green) in ATII^ER:KRASV12^ cells. DAPI (blue) was used to stain nuclei. Scale bars: 40 μm. **b** Immunofluorescence staining of F-actin (red) in ATII^ER:KRASV12^ cells cultured in the absence or presence of 100 ng/ml EGF for 24 h. Rhodamine-phalloidin was used to stain F-actin. DAPI (blue) was used to stain nuclei. Scale bars: 40 μm. **c** Fold change in mRNA levels of *CDH1* (E-cadherin), *VIM* (Vimentin), *SNAI1* (Snail1), *SNAI2* (Snail2), *TWIST*, *ZEB1* and *ZEB2* in ATII^ER:KRASV12^ cells cultured in the absence or presence of 100 ng/ml EGF for 24 h. GAPDH-normalised mRNA levels in control cells were used to set the baseline value at unity. Data are mean ± s.d. *n* = 3 samples per group. **P < 0.01. ***P < 0.001. **d** Protein expression of E-cadherin, ZEB1 and phospho-ERK (p-ERK) in ATII^ER:KRASV12^ treated with 100 ng/ml EGF for 8 or 24 h. β-actin was used as a loading control. **e** Protein expression of E-cadherin, ZEB1, phospho-Smad2 (p-Smad2), phospho-ERK (p-ERK) in primary human ATII cells treated with 100 ng/ml EGF or 5 ng/ml TGFβ over 7 days. β-actin was used as a loading control

**Fig. 2:**
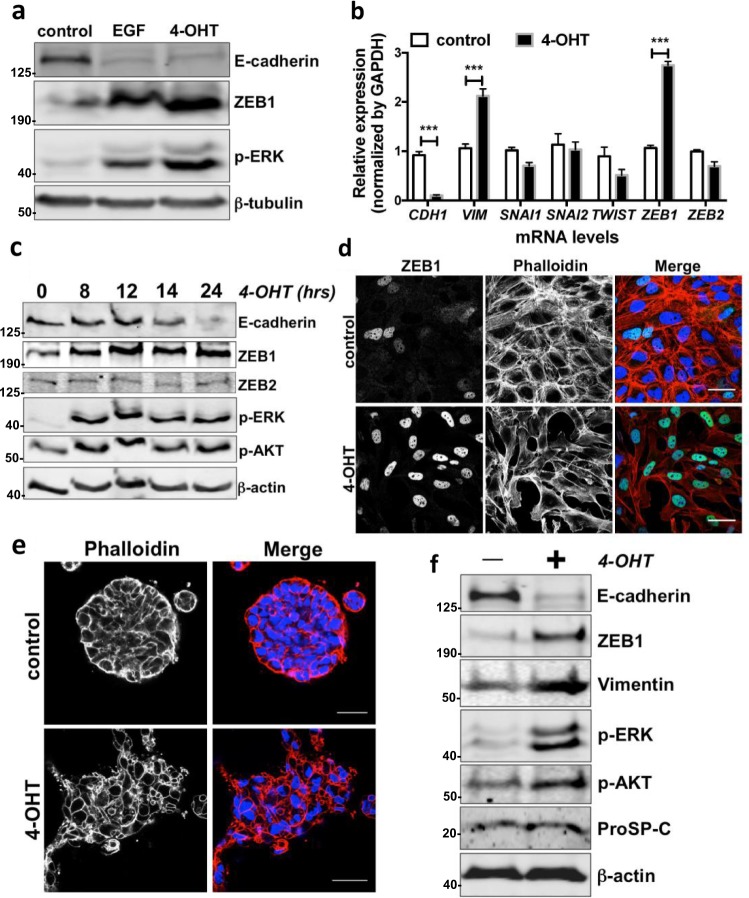
Activation of RAS signalling induces EMT in alveolar epithelial cells. **a** Protein expression of E-cadherin, ZEB1 and phospho-ERK (p-ERK) in ATII^ER:KRASV12^ treated with 100 ng/ml EGF or 250 nM 4-OHT for 24 h. β-tubulin was used as a loading control. **b** Fold change in mRNA levels of *CDH1* (E-cadherin), *VIM* (Vimentin), *SNAI1* (Snail1), *SNAI2* (Snail2), *TWIST*, *ZEB1* and *ZEB2* in ATII^ER:KRASV12^ cells cultured in the absence or presence of 250 nM 4-OHT for 24 h. GAPDH-normalised mRNA levels in control cells were used to set the baseline value at unity. Data are mean ± s.d. *n* = 3 samples per group. ***P < 0.001. **c** Protein expression of E-cadherin, ZEB1, ZEB2, phospho-ERK (p-ERK) and phospho-AKT (p-AKT) in ATII^ER:KRASV12^ treated with 250 nM 4-OHT for the indicated period. β-actin was used as a loading control. **d** Immunofluorescence staining of ZEB1 (green) and F-actin (red) in ATII^ER:KRASV12^ cells cultured in the absence or presence of 250 nM 4-OHT for 24 h. Rhodamine-phalloidin was used to stain F-actin. DAPI (blue) was used to stain nuclei. Scale bars: 40 μm. **e** Representative 3D confocal images of ATII^ER:KRASV12^ cells cultured in Matrigel in the absence or presence of 250 nM 4-OHT for 48 h. Spheres were stained for F-actin with Rhodamine-phalloidin (red) and DAPI (blue). Scale bars: 40 μm. **f** Western blot analysis of lysates from 3D-cultured ATII^ER:KRASV12^ cells in Matrigel treated without or with 250 nM 4-OHT for 48 h showing effects on E-cadherin, ZEB1, Vimentin, phospho-ERK (p-ERK), phospho-AKT (p-AKT) and Pro-surfactant protein-C (Pro-SP-C). β-actin was used as a loading control

Based on the transcriptomic data, we hypothesised an important role of EGFR signalling in IPF. Identification of pathological mechanisms of IPF has been challenging; however, dysregulation of alveolar type 2 (ATII) epithelial cells is thought to be central [5]. We therefore treated a human ATII cell line (ATII^ER:KRASV12^) [17, 18] with EGF (Fig. 1b–d; Supplementary Fig. S1b) or transforming growth factor α (TGFα) (Supplementary Fig. S1b) to activate EGFR signalling. The human ATII cell line grows in continuous culture and expresses the ATII cell marker, pro-surfactant protein C (ProSP-C) (Figs. 1a, 2f). Our results showed that treatment of ATII^ER:KRASV12^ cells with EGF for 24 h induced EMT, reflected by a change in their morphology from typical cuboidal epithelial cells to a more elongated mesenchymal cell phenotype with a reorganisation of the actin cytoskeleton as demonstrated using Phalloidin staining of filamentous actin (F-actin) (Fig. 1b). This phenotypic switch was accompanied by a significant increase in mRNA expression of *ZEB1* and *VIM* (Vimentin), and a reduction in *CDH1* (E-cadherin); mRNA levels of other EMT-TFs, such as *SNAI1*, *SNAI2*, *TWIST* and *ZEB2* were not increased by activation of EGFR signalling (Fig. 1c). The changes in ZEB1 and E-cadherin were further confirmed by Western blot analysis (Fig. 1d; Supplementary Fig. S1b).

Similar results were obtained using primary human ATII cells treated with EGF where an increase in ZEB1 expression was associated with down-regulation of E-cadherin (Fig. 1e). Under the same conditions, however, TGFβ was not able to induce EMT in the primary human ATII cells (Fig. 1e). Together, these results demonstrate that activation of EGFR signalling is able to activate the EMT programme in ATII cells, which is supported by a morphology change, the induction of the EMT-TF ZEB1 and a mesenchymal marker Vimentin as well as a reduction in E-cadherin expression.

### Activation of the RAS pathway drives EMT via ERK–ZEB1 in ATII cells

RAS signalling is one of the most important pathways downstream of EGFR activation and is involved in a variety of physiological and pathological responses, including EMT [19–21]. To investigate whether the RAS pathway is important for EMT in ATII cells, we utilised a RAS-inducible ATII cell model. KRASV12 (containing a single amino acid mutation in *KRAS*, glycine to valine at position 12) fused to the oestrogen receptor (ER) ligand-binding domain [22] was introduced into ATII cells to generate ATII^ER:KRASV12^, in which KRASV12 expression is induced by 4-hydroxytamoxifen (4-OHT) [17, 18]. Like EGF, direct activation of the RAS pathway in ATII^ER:KRASV12^ cells by treatment with 4-OHT induced EMT, reflected by a reduction in E-cadherin levels and an increase in ZEB1 and Vimentin expression (Fig. 2a, b). Time-course analysis further demonstrated that the induction of ZEB1 by RAS activation preceded the down-regulation of E-cadherin (Fig. 2c). Consistently, an EMT morphology change with an increase in ZEB1 expression was observed upon RAS activation (Fig. 2d). When grown on a thick layer of Matrigel, ATII cells form spheres (a 3D culture model) [23]. We adopted this experimental system and used ATII^ER:KRASV12^ cells to investigate whether RAS activation induces EMT in 3D cultures. Control ATII^ER:KRASV12^ cells formed single round spheres. Induction of oncogenic *KRAS* by 4-OHT resulted in spheres invading into the Matrigel with protrusions (Fig. 2e; Supplementary Fig. S2). We recovered these cells from the Matrigel, and examined the protein expression. We confirmed that RAS activation induced EMT in 3D cultures, demonstrated by a reduction in E-cadherin, and an increase in ZEB1 and Vimentin expression (Fig. 2f). These observations suggest that EGFR signalling and the downstream RAS pathway are able to induce EMT in ATII cells.

**Fig. 3:**
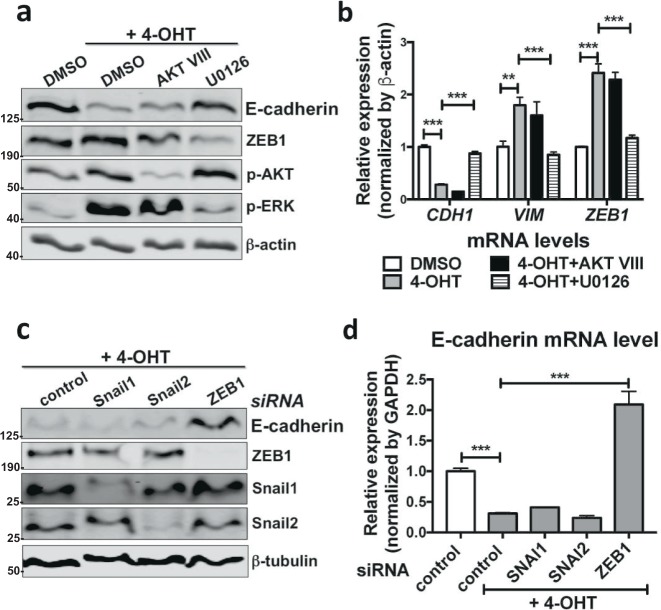
Activation of the RAS pathway drives EMT via ERK–ZEB1 in ATII cells. **a** Protein expression of E-cadherin, ZEB1, phospho-AKT (p-AKT) and phospho-ERK (p-ERK) in ATII^ER:KRASV12^ treated with 250 nM 4-OHT in the absence or presence of inhibitors AKT VIII (10 μM) or U0126 (10 μM) for 24 h. DMSO was used as a vehicle control and β-actin was used as a loading control. **b** Fold change in mRNA levels of *CDH1* (E-cadherin), *VIM* (Vimentin) and *ZEB1* in ATII^ER:KRASV12^ treated with 250 nM 4-OHT in the absence or presence of inhibitors AKT VIII (10 μM) or U0126 (10 μM) for 24 h. DMSO was used as a vehicle control. GAPDH-normalised mRNA levels in control cells were used to set the baseline value at unity. Data are mean ± s.d. *n* = 3 samples per group. **P < 0.01. ***P < 0.001. **c** Protein expression of E-cadherin, ZEB1, Snail1 and Snail2 in ATII^ER:KRASV12^ cells transfected with the indicated siRNA followed by treatment of 250 nM 4-OHT for 24 h. β-tubulin was used as a loading control. **d** Fold change in the mRNA level of *CDH1* (E-cadherin) in ATII^ER:KRASV12^ cells transfected with the indicated siRNA followed by treatment of 250 nM 4-OHT for 24 h. GAPDH-normalised mRNA levels in control cells were used to set the baseline value at unity. Data are mean ± s.d. *n* = 3 samples per group. ***P < 0.001

Since RAS activity regulates both the RAF–ERK and phosphoinositide 3-kinase (PI3K)–protein kinase B (AKT) signalling pathways, we next investigated which one is required for EMT in the ATII cells using inhibitors for these pathways. Treatment with the ERK inhibitor U0126 in ATII^ER:KRASV12^ cells was sufficient to inhibit RAS-induced ZEB1 and Vimentin expression, as well as to restore the expression of E-cadherin and the epithelial morphology; in contrast, the AKT inhibitor AKT VIII failed to do so (Fig. 3a, b; Supplementary Fig. S3a).

We next investigated which EMT-TFs are important for RAS-induced EMT in ATII cells. *ZEB1* RNA interference (RNAi), but not *SNAI1* or *SNAI2* RNAi, was able to restore E-cadherin expression and the epithelial morphology in 4-OHT-treated ATII^ER:KRASV12^ cells (Fig. 3c, d; Supplementary Fig. S3b), in line with the fact the ZEB1 was the only EMT-TF induced by EGFR–RAS signalling (Figs. 1, 2). Taken together, our results identify that RAS activation in human ATII cells drives EMT via ERK–ZEB1 pathway.

### ZEB1 is highly expressed in IPF alveolar epithelium and is critical for transcriptional regulation of secreted factors that mediate crosstalk between ATII cells and fibroblasts

**Fig. 4:**
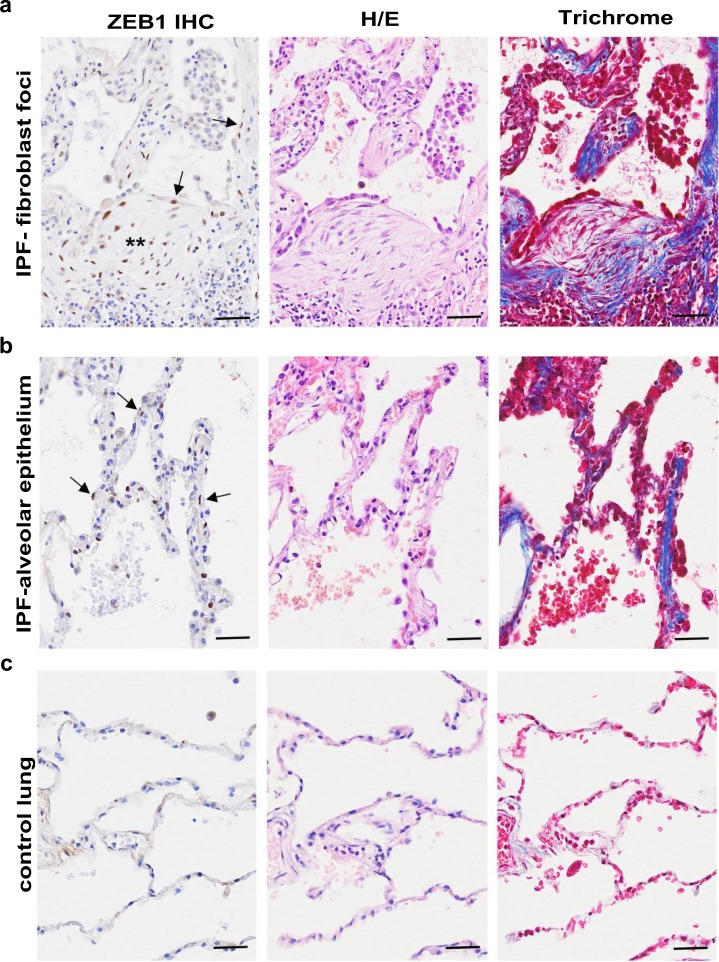
ZEB1 is highly expressed in IPF fibroblastic foci and epithelial cells of thickened alveoli septae where collagen deposition in the interstitium is also evident. Serial sections of IPF (fibroblastic foci in **a** and epithelial cells of thickened alveoli septae in **b**) or control lung tissue (**c**) were stained for ZEB1 (left panel), with H/E (middle panel) or Masson’s trichrome stain (right panel, collagen shown in blue). *n* = 3. Arrows: representative positive ZEB1 staining. **—a fibroblastic focus. Scale bars: 50 μm

Given our in vitro findings, we compared ZEB1 expression in IPF and control lung tissue. In IPF tissue, we detected strong nuclear expression of ZEB1 not only in fibroblastic foci (Fig. 4a) but also in epithelial cells of thickened alveoli septae where collagen deposition in the interstitium was evident (Fig. 4b); in contrast, little ZEB1 staining or collagen deposition was observed in alveoli of control lung tissue (Fig. 4c). The presence of nuclear ZEB1 staining in alveolar epithelial cells within IPF lung tissue suggests that these cells are undergoing EMT; furthermore, the presence of ECM suggests induction of mesenchymal responses, either directly via the repairing epithelial cells undergoing EMT or by crosstalk with underlying fibroblasts.

**Fig. 5:**
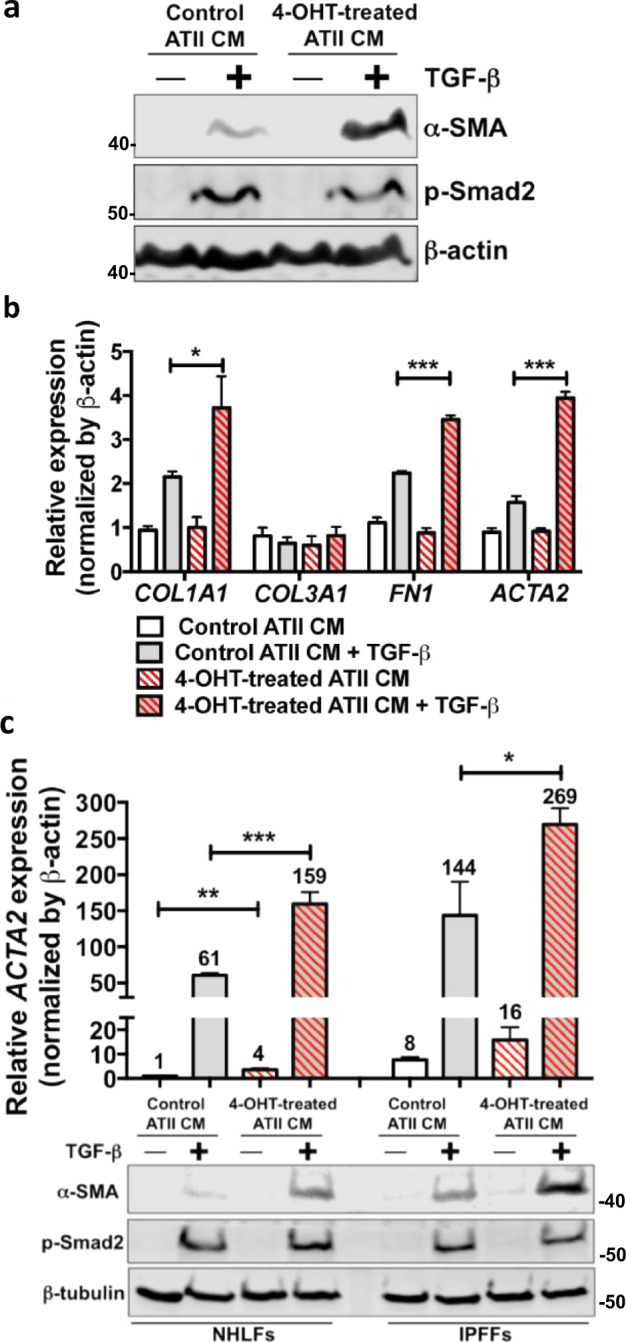
ATII cells undergoing RAS-induced EMT induce fibroblast activation via paracrine signalling. **a** Protein expression of α-SMA and phospho-Smad2 (p-Smad2) in MRC5 lung fibroblasts treated without or with 5 ng/ml TGFβ in the presence of conditioned media (CM) from control or 4-OHT-treated ATII^ER:KRASV12^ cells for 48 h. β-actin was used as a loading control. **b** Fold change in mRNA levels of *COL1A1*, *COL3A1, FN1* and *ACTA2* in MRC5 lung fibroblasts with indicated treatments. β-actin-normalised mRNA levels in control cells were used to set the baseline value at unity. Data are mean ± s.d. *n* = 3 samples per group. *P < 0.05. ***P < 0.001. **c** Protein expression of α-SMA and phospho-Smad2 (p-Smad2), and fold change in the mRNA level of *ACTA2* (α-SMA) in primary human lung fibroblasts from IPF (IPFFs) or from normal healthy lung (NHLFs) with indicated treatments. β-tubulin was used as a loading control in Western blots. β-actin-normalised mRNA levels in control cells were used to set the baseline value at unity (indicated above bars). Data are mean ± s.d. *n* = 3 samples per group. *P < 0.05. **P < 0.01. ***P < 0.001

Comparison of the relative expression of ECM components in RAS-activated ATII^ER:KRASV12^ cells and fibroblasts highlights that ATII cells produce extremely low levels of ECM genes even after the induction of EMT (Supplementary Fig. S4a), suggesting that ECM production in fibrosis is more likely to be a consequence of fibroblast activation than direct deposition by epithelial cells undergoing EMT. Therefore we investigated whether ATII cells undergoing RAS-induced EMT produce paracrine factors that activate fibroblasts. For these experiments, we took advantage of the ability of 4-OHT to induce RAS pathway activation in ATII^ER:KRASV12^ cells, as this was not dependent on exogenous growth factors that might directly affect fibroblast responses. We treated the MRC5 or primary human parenchymal lung fibroblasts with conditioned media (CM) from control or 4-OHT-treated ATII^ER:KRASV12^ cells in the absence or presence of TGFβ, and evaluated the fibroblast responses by measuring the expression of α-smooth muscle actin (α-SMA, a myofibroblast marker) and other ECM genes, including *COL1A1*, *COL3A1* and *FN1*. On its own, CM from RAS-activated ATII^ER:KRASV12^ cells (4-OHT-treated ATII CM) had little effect on the activation of fibroblasts (Fig. 5). However, 4-OHT-treated ATII CM together with TGFβ achieved a synergistic effect in activating fibroblasts, reflected by a larger increase in α-SMA (*ACTA2*), *COL1A1* and *FN1* levels (Fig. 5a, b). Of note, 4-OHT-treated ATII CM did not augment Smad2 phosphorylation suggesting a Smad2-independent response (Fig. 5a, c). Similar results were obtained using primary human lung fibroblasts from IPF patients (IPF fibroblasts, IPFFs) and control donors (normal human lung fibroblasts, NHLFs) (Fig. 5c; Supplementary Fig. S4b).

**Fig. 6:**
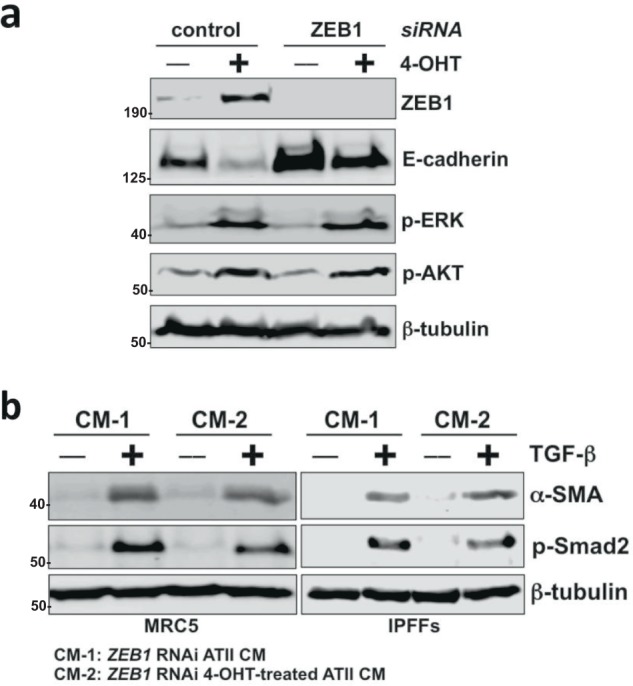
ZEB1 is a key regulator of the paracrine signalling between ATII cells and fibroblasts. **a** Protein expression of ZEB1, E-cadherin, phospho-ERK (p-ERK) and phospho-AKT (p-AKT) in ATII^ER:KRASV12^ cells with indicated treatments. β-tubulin was used as a loading control. **b** Protein expression of α-SMA and phospho-Smad2 (p-Smad2) in MRC5 lung fibroblasts or primary human lung fibroblasts from IPF (IPFFs) with indicated treatments. β-tubulin was used as a loading control

Given the important role of ZEB1 in mediating RAS-induced EMT and the fact that ZEB1 is highly expressed in the alveolar epithelium of IPF patients, we hypothesised that ZEB1 may determine the paracrine signalling produced by ATII cells undergoing RAS-induced EMT. *ZEB1* RNAi (Fig. 6a) in ATII cells completely abolished the effects of CM from RAS-activated ATII cells on TGFβ-induced activation of fibroblasts (Fig. 6b; Supplementary Fig. S5), highlighting ZEB1 as a key regulator of EMT as well as the paracrine signalling between ATII cells and fibroblasts.

### ZEB1 regulates the expression of tissue plasminogen activator, which acts as a paracrine regulator of TGFβ-induced fibroblast activation

**Fig. 7:**
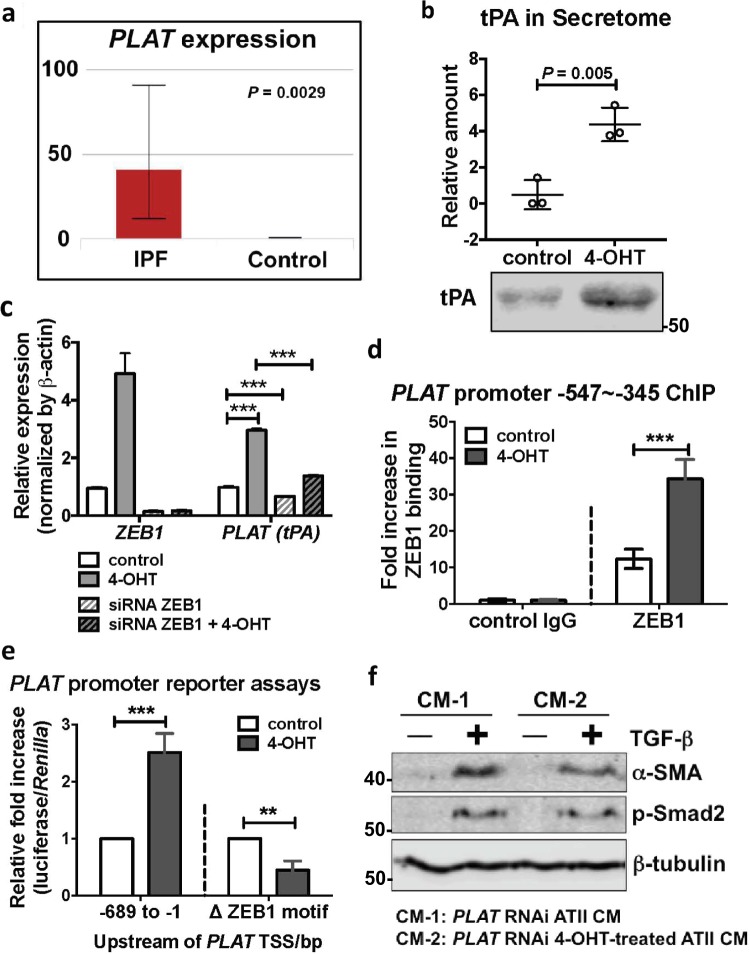
ZEB1 regulates the expression of tissue plasminogen activator (tPA), which acts as a paracrine regulator of TGFβ-induced fibroblast activation. **a** Increased expression of *PLAT* (tPA) in IPF epithelial cells is shown by an online LGEA web portal (https://research.cchmc.org/pbge/lunggens/mainportal.html). **b** Quantitative secretome analysis identifies an increased level of tPA in the conditioned media (CM) from 4-OHT-treated ATII^ER:KRASV12^ cells and a representative tPA Western blot of CM from control or 4-OHT-treated ATII^ER:KRASV12^ cells. Data are individual values with mean and s.d. *n* = 3 samples per group. Values were normalised to total fmol of each sample multiplied by 10,000. **c** Fold change in mRNA levels of *ZEB1* and *PLAT* (tPA) in ATII^ER:KRASV12^ cells with indicated treatments. β-actin-normalised mRNA levels in control cells were used to set the baseline value at unity. Data are mean ± s.d. *n* = 3 samples per group. ***P < 0.001. **d** ChIP assays of ZEB1's ability to bind the *PLAT* (tPA) promoter in ATII^ER:KRASV12^ cells with indicated treatments. The amplified *PLAT* (tPA) promoter region (−547 to −345) contains a ZEB1 binding site at −419. Values represent relative binding in relation to input (2%), normalised against control (1.0). Data are mean ± s.d. *n* = 4 samples per group. ***P < 0.001. **e** *PLAT* promoter reporter assays in ATII^ER:KRASV12^ cells with indicated treatments. Values represent relative fold of firefly luciferase in relation to *Renilla* luciferase, normalised against control (1.0). Data are mean ± s.d. *n* = 3 samples per group. **P < 0.01. ***P < 0.001. **f** Protein expression of α-SMA and phospho-Smad2 (p-Smad2) in MRC5 lung fibroblasts with indicated treatments. β-tubulin was used as a loading control

By performing quantitative proteomic analysis of the CM from control or 4-OHT-treated ATII^ER:KRASV12^ cells, we identified ~430 secreted proteins whose levels changed during RAS-induced EMT. We then checked their expression in pulmonary epithelial cells from control and IPF lung tissue using a publicly available dataset [24], and identified a total number of 25 genes/proteins that were elevated in IPF lung epithelial cells as well as in CM from 4-OHT-treated ATII^ER:KRASV12^ cells (Supplementary Table S1). Of these, *PLAT*, which encodes tPA was most up-regulated in IPF epithelial cells (Fig. 7a; Supplementary Table S1) and we confirmed enhanced secretion of tPA in the CM from 4-OHT-treated ATII^ER:KRASV12^ cells by Western blotting (Fig. 7b). As we had identified ZEB1 as the key regulator of epithelial–mesenchymal crosstalk, we scanned the promoter of *PLAT* for the presence of ZEB1 binding motifs (5′-CANNTG-3′) and found a ZEB1 binding site −419 bp upstream of the transcriptional start site (TSS) (Supplementary Fig. S6a). Further experiments showed that the mRNA expression of *PLAT* was increased upon RAS-activation in ATII cells and this was repressed by *ZEB1* RNAi (Fig. 7c).

To validate the ZEB1 binding site in the *PLAT* promoter, we first performed a chromatin immunoprecipitation (ChIP) assay. An anti-ZEB1 antibody was used to precipitate formaldehyde cross-linked ZEB1-DNA complexes in ATII^ER:KRASV12^ cells treated without or with 4-OHT. The presence of *PLAT* promoter DNA sequences in the immunoprecipitate was verified by PCR using primers amplifying the region between −547 and −345 upstream of the TSS, and we found RAS activation in ATII cells increased ZEB1 occupancy on the *PLAT* promoter (Fig. 7d; Supplementary Fig. S6b). We next generated two *PLAT* promoter constructs (−689 to −1 upstream of the TSS) which were cloned into a pGL3 basic luciferase reporter plasmid and transfected into ATII cells; the pGL3 basic*-PLAT* (−689 to −1) construct contained the ZEB1 motif whereas this was deleted in the second construct (delta −419 to −414 upstream of the TSS) (pGL3 basic-Δ ZEB1 motif). RAS activation by 4-OHT in ATII^ER:KRASV12^ cells resulted in a significant increase in pGL3 basic*-PLAT* (−689 to −1) luciferase activity. Under the same conditions, luciferase activity was not increased using pGL3 basic-Δ ZEB1 motif (Fig. 7e). These data confirm that *PLAT* (tPA) is a transcriptional target of ZEB1 in response to RAS activation in ATII cells.

Consistent with a previous report [25], tPA synergistically promoted TGFβ-induced α-SMA expression in human lung fibroblasts (Supplementary Fig. S6c). Like ZEB1, *PLAT* RNAi (Supplementary Fig. S6d) in ATII cells completely abolished the effects of CM from RAS-activated ATII cells on TGFβ-induced α-SMA expression in fibroblasts (Fig. 7f), demonstrating tPA as a key paracrine factor secreted by ATII cells undergoing RAS-induced EMT. These results provide clear evidence that a ZEB1-tPA axis is involved in the paracrine signalling between ATII cells undergoing RAS-induced EMT and fibroblasts to augment their differentiation into myofibroblasts caused by TGFβ.

Finally, in view of the requirement for exogenous TGFβ to demonstrate an effect of the 4-OHT-treated ATII CM on fibroblasts, we investigated whether ATII cells in fibrotic tissue in vivo or those undergoing injury/repair in vitro expressed endogenous TGFβ. Using a publicly available dataset [24], we found that the major *TGFB* isoform expressed by alveolar epithelial cells in vivo was *TGFB2* and that this was expressed at significantly higher levels in IPF compared with control lung tissue (Supplementary Fig. S7a). In contrast with the study in kidney [14], the data also revealed that Snail2 is up-regulated in IPF vs. control lung epithelial cells, but not Snail1 or Twist (Supplementary Fig. S7b). As we have previously shown that scrape-wounding of bronchial epithelial cells stimulates release of TGFβ2 independently of EGFR activation [26], we examined whether damage of ATII cells similarly affected *TGFB2* expression. This showed that scrape-wounded ATII cells expressed more *TGFB2* and this increased in proportion to the extent of injury (Supplementary Fig. S7c). These data suggest that damaged ATII cells are a potential a source of TGFβ in vivo.

## Discussion

**Fig. 8:**
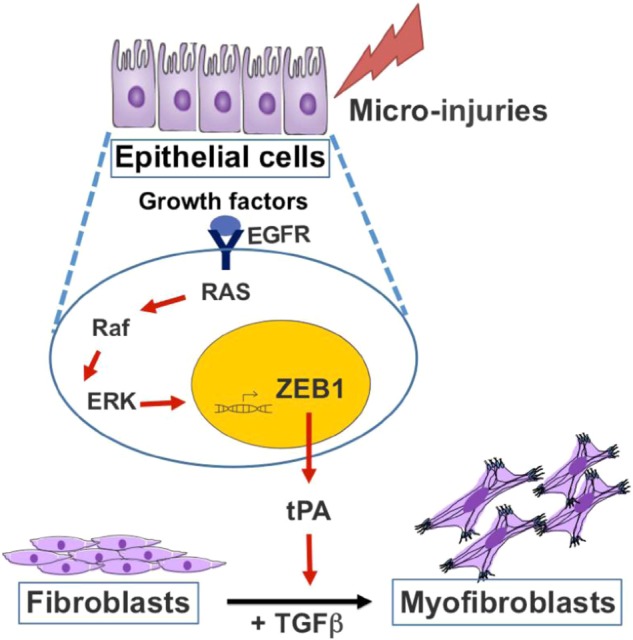
Diagram summarising a critical role of ZEB1-tPA axis regulated by EGFR–RAS–ERK pathway in the development of lung fibrosis (details provided in Discussion)

Fibrotic diseases are a major cause of morbidity and mortality worldwide and their prevalence is increasing with an ageing population. Abnormal wound healing responses appear to make major contributions to the scarring process, but the underlying pathological mechanisms are unclear, especially the role of EMT. In this study, we have used a variety of approaches to show that activation of EGFR–RAS–ERK signalling in ATII cells induces EMT via the transcriptional regulator ZEB1. Importantly, beyond its effects on the epithelial cell phenotype, we have identified that ZEB1 is a regulator of paracrine signalling between lung epithelial cells and fibroblasts, as ATII cells undergoing RAS-induced EMT secrete tPA to augment TGFβ-induced myofibroblast differentiation (Fig. 8). This may be an important profibrotic event as, relative to epithelial cells, the ability of fibroblasts to synthesise ECM is orders of magnitude greater.

Consistent with previous findings [27, 28], we found strong expression of ZEB1 in the epithelium in proximity to fibroblastic foci in IPF lung tissue. However, we also found ZEB1 was expressed in epithelial cells of thickened alveolar septae where ECM deposition was evident. This suggests that ZEB1 is induced as an early response to alveolar epithelial injury and that, by regulating the expression of factors involved in paracrine signalling, ZEB1 may promote TGFβ-induced fibroblast activation in IPF. While this may be a normal physiological response to injury, persistent epithelial injury and/or failure to resolve the lesion may sensitise the underlying fibroblasts to drive a pathologic profibrogenic response. In line with this, exposure of human lung cells to nickel (Ni), an environmental and occupational pollutant linked to lung fibrosis [29], caused ZEB1-dependent EMT, which was irreversible even after the termination of Ni exposure [30]. Thus, it is conceivable that repetitive environmental exposures to metals such as Ni could lead to deregulation of ZEB1 to cause persistent EMT and exaggerated profibrogenic crosstalk during the initiation of IPF.

EMT in the ATII cells was strongly induced by EGFR activation. The EGFR is a transmembrane receptor tyrosine kinase activated by members of the EGF family, including EGF and TGFα [31]. EGFR dimerisation activates one or more downstream effectors including the ERK, PI3K/AKT, signal transducer and activator of transcription (STAT) and mammalian target of rapamycin (mTOR) pathways through receptor autophosphorylation and cytoplasmic protein binding [32–34]. These in turn act as critical mediators of airway and alveolar homoeostasis, with aberrant activation within one or more pathway components capable of driving a variety of respiratory pathologies, including lung fibrosis [33, 35]. The EGFR pathway has been implicated in lung fibrosis through studies in which transgenic mice that constitutively express TGFα in epithelial cells develop progressive lung fibrosis [36, 37]. Conversely, mice deficient in TGFα that lack normal EGFR signalling or that are treated with EGFR pathway inhibitors exhibit resistance to bleomycin-induced lung fibrosis [38]. In IPF patients, *EGFR* mutations [39] or increased expression of the EGFR [40] have been reported. Our evidence that an EGFR–RAS–ERK–ZEB1 axis may contribute to the early stages of lung fibrosis suggests that inhibiting EGFR signalling may be of clinical relevance for regulating human fibrotic lung disease.

A key finding of our study was identification that ZEB1 controls tPA expression and that this affects the sensitivity of fibroblast activation induced by TGFβ. While tPA is a key activator of fibrinolysis, it also has direct cellular effects by virtue of its ability to bind to the low-density lipoprotein (LDL) receptor-related protein-1 (LRP-1), triggering LRP-1 tyrosine phosphorylation and recruitment of β1-integrin signalling involving integrin-linked kinase (ILK) [25]. In this context, tPA acts as a survival factor that protects fibroblasts/myofibroblasts from apoptosis, and it has previously been implicated in kidney fibrosis [25], but no other fibrotic conditions. In keeping with a role of tPA in human lung fibrosis was the identification in a publicly available dataset [24] that there is an increased expression of *PLAT* (tPA) by IPF epithelial cells.

EMT converts epithelial cells into migratory and/or invasive mesenchymal cells, and is well established during development and carcinogenesis, however, its role in fibrosis has been more controversial [1, 3, 41]. Although human IPF tissue studies have demonstrated co-localisation of epithelial and mesenchymal markers [27, 28, 42], the number of fibroblasts arising from epithelial cells was small in some mouse lineage tracing studies [11, 12], suggesting effects of EMT beyond direct phenotypic conversion into matrix-producing cells. Recent studies in renal fibrosis have reported that tubular epithelial cells undergoing EMT relay signals to the interstitium, which promote myofibroblast differentiation and fibrogenesis, without directly contributing to the myofibroblast population [14, 43–45]. In tubulointerstitial renal fibrosis, TGFβ induces EMT via Snail1, and then Snail1 induces TGFβ expression generating an autocrine loop that sustains the progression of the disease by influencing the differentiation of fibroblasts into myofibroblasts [14]. In contrast with studies in kidney, our analysis of publicly available transcriptomic datasets of IPF lung tissue, identified the EGFR–ERK pathway as the top-ranked pathway with 150 of 458 pathway candidates being significantly overrepresented in the IPF dataset, highlighting the potential importance of this pathway in IPF pathogenesis. Building on these observations, our in vitro studies of EGFR–RAS–ERK-induced EMT, identified ZEB1 as the main transcription factor that controlled EMT as well as paracrine signalling through regulation of tPA expression, which potentiated fibroblast differentiation in the presence of TGFβ. However, as these paracrine effects required exogenous TGFβ, this raised the question of the source of TGFβ in lung fibrosis in vivo. While many cell types produce TGFβ isoforms which can also be stored as latent growth factor bound in the ECM [46–48], we focussed on the epithelium and found increased expression of *TGFB2*, as well as *SNAI2* in IPF epithelial cells using publicly available datasets, with scrape wounding of ATII cells also inducing *TGFB2* expression in vitro. The increased epithelial *TGFB2* signature highlights the potential for EGF (ZEB1) and TGFβ (Snail2) to synergize in paracrine activation of the underlying fibroblasts.

Together with previous findings in kidney fibrosis [14, 43], our study helps to provide a unified concept for the role of EMT in fibrosis: persistent EMT of epithelial cells may dysregulate paracrine signalling between epithelial and mesenchymal cells, so creating a profibrogenic microenvironment which leads to the development of fibrosis. Based on the relative low levels of ECM biosynthesis by epithelial cells and the relatively small numbers of mesenchymal cells identified in lineage tracing studies [11, 12, 14, 43], this mechanism may be more important than direct conversion of epithelial cells into mesenchymal cells. While the finer details of these paracrine mechanisms may vary according to disease and tissue location, our identification of ZEB1 as a key regulator of EGF/RAS-induced EMT and an enhancer of paracrine signalling mediating the crosstalk between ATII cells and lung fibroblasts may help to find drug targets or biomarkers to intervene or predict the progression of pulmonary fibrosis.

## Methods

### Lung tissue sampling

All human lung experiments were approved by the Southampton and South West Hampshire and the Mid and South Buckinghamshire Local Research Ethics Committees, and all subjects gave written informed consent. Clinically indicated IPF lung biopsy tissue samples and non-fibrotic control tissue samples (macroscopically normal lung sampled remotely from a cancer site) were deemed surplus to clinical diagnostic requirements. All IPF samples were from patients subsequently receiving a multidisciplinary diagnosis of IPF according to international consensus guidelines [49].

### Cell culture, reagents and transfections

Primary parenchymal lung fibroblast cultures were established from IPF or control lung tissue as described previously [50]. Fibroblasts were cultured in Dulbecco’s Modified Eagle’s Medium (DMEM) supplemented with 10% foetal bovine serum (FBS), 50 units/ml penicillin, 50 μg/ml streptomycin, 2 mM L-glutamine, 1 mM sodium pyruvate and 1× non-essential amino acids (DMEM/FBS) (all from Life Technologies).

Primary human ATII cells were isolated from macroscopically normal regions of surgically resected lung parenchyma as described previously [50, 51]. The alveolar epithelial cells were resuspended in fresh DCCM-1 (Biological Industries Ltd.) supplemented with 10% new-born calf serum (NBCS) (Life Technologies), 1% penicillin, 1% streptomycin and 1% L-glutamine (all from Sigma Aldrich) and plated on collagen 1 (PureCol 5005-b, Advanced BioMatrix Inc.) coated 96 well plates at 60% density; purity of the cultures was determined by staining for alkaline phosphatase.

ATII^ER:KRASV12^ cells [17, 18] were cultured in DCCM-1 (Biological Industries Ltd.) supplemented with 10% NBCS (Life Technologies), 1% penicillin, 1% streptomycin and 1% L-glutamine (all from Sigma Aldrich). To induce RAS activation in ATII^ER:KRASV12^ cells, 250 nM 4-OHT (Sigma-Aldrich) was added [17, 18]. MRC5 cells were obtained from the European Collection of Authenticated Cell Cultures (ECACC) and were cultured in DMEM (Thermo Fisher Scientific). Both cell culture media were supplemented with 10% FBS (Thermo Fisher Scientific), 1% penicillin/streptomycin and glutamine (Thermo Fisher Scientific). All cells were kept at 37 °C and 5% CO_2_. For 3D culture, ATII^ER:KRASV12^ cells were cultured as previously described [23] in Matrigel (BD Biosciences). TGFα was from Fisher Scientific UK Ltd. TGFβ1 was from PeproTech. EGF and recombinant human tPA protein were from Bio-Techne. AKT VIII and U0126 were from Sigma Aldrich. No mycoplasma contamination was detected in the cell lines used.

Short interfering RNA (siRNA) oligos against *ZEB1* (MU-006564-02-0002), *SNAI1* (Snail1) (MU-010847-00-0002), *SNAI2* (Snail2) (MU-017386-00-0002) and *PLAT* (tPA) (MU-005999-01-0002) were purchased from Dharmacon. Sequences are available from Dharmacon, or on request. As a negative control, we used siGENOME RISC-Free siRNA (Dharmacon). ATII^ER:KRASV12^ cells were transfected with the indicated siRNA oligos at a final concentration of 35 nM using DharmaFECT 2 reagent (Dharmacon).

### Western blot analysis

Western blot analysis was performed with lysates from cells with urea buffer (8 M urea, 1 M thiourea, 0.5% CHAPS, 50 mM DTT and 24 mM Spermine). Primary antibodies were from Santa Cruz (β-actin, sc-47778; ZEB1, sc-25388; ZEB2, sc-48789; E-cadherin, sc-21791; Snail2, sc-10436), Abcam (β-tubulin, ab6046), Cell Signalling Technology (α-SMA, 14968; phospho-AKT, 9271; phospho-ERK, 9101; Snail1, 3879; Snail2, 9585; TWIST, 46702; Phospho-Smad2, 3104; β-tubulin, 86298), BD Transduction Laboratories (E-cadherin, 610405; Vimentin, 550513) and Millipore (proSP-C, AB3786; tPA, 05-883). Signals were detected using an Odyssey imaging system (LI-COR), and evaluated by ImageJ 1.42q software (National Institutes of Health).

### qRT-PCR

Total RNA was isolated using RNeasy mini kit (Qiagen) according to manufacturer’s instructions and quantified using a Nanodrop Spectrophotometer 2000c (Thermo Fisher Scientific). Real-time quantitative RT-PCR was carried out using gene-specific primers (QuantiTect Primer Assays, Qiagen) for *CDH1* (E-cadherin) (QT00080143), *SNAI1* (Snail1) (QT00010010), *SNAI2* (Snail2) (QT00044128), *ZEB1* (QT00008555), *ZEB2* (QT00008554), *TWIST* (QT00011956), *VIM* (QT00095795), *COL1A1* (QT00037793), *COL3A1* (QT00058233), *FN1* (QT00038024), *ACTA2* (α-SMA) (QT00088102), *PLAT* (tPA) (QT00075761), *TGFB1* (QT00000728), *TGFB2* (QT00025718), *GAPDH* (QT01192646) or *ACTB* (β-actin) (QT01680476) with QuantiNova SYBR Green RT-PCR kits (Qiagen). Relative transcript levels of target genes were normalised to *GAPDH* or *ACTB* (β-actin).

### Immunofluorescence microscopy

Cells were fixed in 4% PBS-paraformaldehyde for 15 min, incubated in 0.1% Triton X-100 for 5 min on ice, then in 0.2% fish skin gelatin in PBS for 1 h and stained for 1 h with an anti-Prosurfactant Protein C (proSP-C) antibody (1:100, Millipore AB3786, rabbit polyclonal) or anti-ZEB1 (1:100, Santa Cruz sc-25388, rabbit polyclonal). Protein expression was detected using Alexa Fluor (1:400, Molecular Probes) for 20 min. DAPI (Invitrogen) was used to stain nuclei (1:1000). Rhodamine-phalloidin was used to visualise filamentous actin (F-actin) (Molecular Probes). For immunofluorescence staining of 3D cultures from ATII^ER:KRASV12^ cells, spheres were fixed with 4% PBS-paraformaldehyde for 40 min, permeabilised in 0.5% Triton X-100 for 10 min on ice and stained with rhodamine-phalloidin for 1 h at room temperature. Spheres were counterstained with DAPI. Samples were observed using a confocal microscope system (Leica SP8). Acquired images were analysed using Photoshop (Adobe Systems) according to the guidelines of the journal.

### Immunohistochemistry, haematoxylin and eosin (H/E) and tinctorial stains

Control or IPF lung tissues (*n* = 3 donors) were fixed and embedded in paraffin wax; tissue sections (4 µm) were processed and stained as previously described [20]. Briefly, the tissue sections were de-waxed, rehydrated and incubated with 3% hydrogen peroxide in methanol for 10 min to block endogenous peroxidase activity. Sections were then blocked with normal goat serum and incubated at room temperature with a primary antibody against ZEB1 (1:500, Sigma), followed by a biotinylated secondary antibody (1:500, Vector Laboratories Ltd., UK); antibody binding was detected using streptavidin-conjugated horse-radish peroxidase and visualised using DAB (DAKO) before counterstaining with Mayer’s Haematoxylin. For H/E stain, Shandon Varistain 24-4 automatic slide stainer (Thermo Fisher Scientific) was used. For tinctorial stain, Trichrome stain (Abcam ab150686) was used according to the manufacturers’ instructions. Images were acquired using an Olympus Dotslide Scanner VS110.

### Chromatin immunoprecipitation (ChIP)

ChIP assays were carried out using SimpleChIP enzymatic chromatin IP kits (Cell Signalling Technology) as per the manufacturer’s instructions. Briefly, ATII^ER:KRASV12^ cells with indicated treatments were incubated for 10 min with 1% formaldehyde solution at room temperature, followed by incubation with 125 mM glycine. Antibodies used for ChIP were as follows: rabbit anti-ZEB1 (PA5-28221, Invitrogen, rabbit polyclonal, 5 μg per IP sample), normal rabbit IgG (2729, Cell Signalling Technology, 5 μg per IP sample). For the ZEB1 binding site at position −419 of the human *PLAT* (tPA) promoter, the primers amplifying the region between −547 and −345 were as follows: forward 5′-GGAAAGTCCCCGGAGGCCACCTA-3′ and reverse 5′-TGGAACACTTTGTGTGGTGGC-3′. DNA fragments were quantified by qPCR. PCR products were analysed in a 1.5% agarose gel by ethidium bromide staining.

### Luciferase constructs and luciferase reporter assays

The human *PLAT* (tPA) promoter (sequence −689 to −1 upstream of the TSS) was amplified from human genomic DNA by PCR, and was subsequently cloned into pGL3 basic vector (Promega), termed pGL3 basic*-PLAT* (−689 to −1). The putative ZEB1 binding site, positioned −419 to −414 on the human *PLAT* promoter, was removed from pGL3 basic*-PLAT* (−689 to −1) construct to create the pGL3 basic-Δ ZEB1 motif construct.

For the luciferase reporter assays, ATII^ER:KRASV12^ cells were transfected using Lipofectamine 3000 (Invitrogen) with 80 ng of phRL-CMV (Promega), which constitutively expresses the *Renilla* luciferase reporter, plus 600 ng of pGL3 basic*-PLAT* (−689 to −1) or pGL3 basic-Δ ZEB1 motif per well in the presence or absence of 4-OHT. Finally, the transcriptional assay was carried out using the Dual-Luciferase reporter assay system (Promega) following the manufacturer’s protocol.

### Quantitative proteomic analysis of the secretome and the subsequent data analysis

Serum-free CM from ATII^ER:KRASV12^ cells treated without or with 4-OHT (250 nM, 24 h) were analysed using an enrichment strategy based upon Strataclean resin (Agilent) in combination with the quantitative label-free approach, LC-MS^E^, to provide in-depth proteome coverage and estimates of protein concentration in absolute amounts [52] (details provided in Supplementary Methods).

Raw data were processed and collated into a single.csv document. Values were then normalised to total fmol of each sample multiplied by 10,000. Pseudo-counts were applied to the normalised values to replace missing ones, to allow for full statistical analysis to be completed [53]. We first sorted the normalised values in each column in order of abundance, in ascending order, then the minimum value of each sample identified. This minimum was used to replace all missing values in the data set. A two-tailed, unpaired Student’s *t*-test was used to compare two groups for independent samples. *P* < 0.05 was considered statistically significant.

In order to highlight their implications in IPF, differentially expressed proteins/genes identified in the quantitative secretome analysis were searched in LGEA web portal (https://research.cchmc.org/pbge/lunggens/mainportal.html) for their levels in pulmonary epithelial cells from control and IPF lung tissue.

### Bioinformatics

IPF transcriptomic data was downloaded from the NCBI’s Gene Expression Omnibus (GEO). We used data from GSE24206 [15], a microarray study comparing samples from 11 IPF patients undergoing lung transplantation or diagnostic biopsy to six normal lung samples taken from lung transplantation donors. Microarray series matrix files were imported into R, and differential expression analysis comparing normal to IPF samples performed using the R package limma [54]. Data were log-transformed before analysis. To correct for multiple testing, a Benjamini–Hochberg FDR of 5% was applied to the data, and a *Q*-value cut-off of 0.02 was used to determine significance. Differentially expressed gene lists were input into the human Consensus Pathways Database, which determined pathways with differentially expressed genes overrepresented in this database. A 5% FDR was used as above.

### Statistical analysis and repeatability of experiments

Each experiment was repeated at least twice. Unless otherwise noted, data are presented as mean and s.d., and a two-tailed, unpaired Student’s *t*-test was used to compare two groups for independent samples. *P* < 0.05 was considered statistically significant.

## Electronic supplementary material

Supplementary Text

Supplementary Fig. S1

Supplementary Fig. S2

Supplementary Fig. S3

Supplementary Fig. S4

Supplementary Fig. S5

Supplementary Fig. S6

Supplementary Fig. S7

Supplementary Table S1
