# Supplementary material for: Paracrine signalling during ZEB1-mediated epithelial–mesenchymal transition augments local myofibroblast differentiation in lung fibrosis
Source: Cell Death Differ. 2018 Jul 26;26(5):943–57. doi: 10.1038/s41418-018-0175-7 (PMC6252080; doi:10.1038/s41418-018-0175-7)
Supplement: Supplementary file 1 — Supplementary Text [file 41418_2018_175_MOESM1_ESM.pdf]

Supplementary Methods

Quantitative proteomic analysis of the secretome.

1. Sample preparation. Serum-free conditioned media (CM) were centrifuged at 17,000 x *g* for 1 min and the supernatants were transferred to fresh tubes. StrataClean resin was vortexed and 50 µL of the slurry was added to 1 mL of the CM and incubated for 1 h at 4°C on a rotator. Samples were centrifuged at 17,000 x *g*, for 3 min and the supernatant removed and discarded. The resin/protein pellet was re-suspended in 300 µL of 100 mM ammonium bicarbonate containing 0.1% SDS. Samples were reduced using 0.5 µg of dithiothreitol and incubating for 1 h with mixing using an Eppendorf Thermomixer® set to 60°C at 1,000 RPM. After 1 h, proteins were alkylated by addition of 2.5 µg iodoacetamide and then incubated for 45 min at room temperature in the dark. Samples were subsequently digested using 0.5 µg sequencing grade modified trypsin (1/50 (w/w)) overnight at 37°C. Digested samples were centrifuged at 17,000 x *g* for 3 min and the peptide supernatant was transferred to fresh tubes. The resin was washed with a further 500 µL of 100% acetonitrile, vortexed, centrifuged at 17,000 x *g*, and the supernatant was removed. The supernatants were combined and lyophilised to dryness at 35°C in a vacuum centrifuge (Eppendorf, UK).

Peptides were reconstituted in 150 µL 3% acetonitrile + 0.1% formic acid and vortexed. Samples were acidified to pH < 3.0 with TFA and loaded onto an Empore C18 96-well solid phase extraction plate (3M, Maplewood, MN). Each acidified sample was loaded onto a conditioned C18 reverse-phase Empore Plate, and washed with 20 µL of 0.5% acetic acid. Peptides were eluted from the plate using 40 µL of 80% acetonitrile + 0.5% acetic acid. Samples were lyophilised to dryness at 35°C and

re-suspended in 25 µL of 2% acetonitrile + 0.1% formic acid containing 100 fmol/µL of the internal enolase digestion standard (Waters).

2. LC-MS^E analysis. 1 µL of digested secretome samples were loaded onto a reverse phase trap column (Symmetry C18, 5 µm, 180 µm x 20mm, Waters Corporation, Milford, MA), at a trapping rate of 5 µL/min and washed for 10 min with buffer A prior to the analytical nanoscale LC separation using a C18 reversed phase column (HSS T3, 1.8 µm, 500 mm x 75µm, Waters). The peptides were eluted over a 120 min linear gradient from 1% acetonitrile + 0.1% formic acid to 60 % acetonitrile + 0.1% formic acid, at a flow rate of 300 nL/min. Eluted samples were sprayed directly into a Synapt G2-Si mass spectrometer (Waters Corporation, Wilmslow, UK) operating in the data independent High Definition Mass Spectometry (HDMS^E) mode. Data were acquired from 50 to 2000 *m/z* using alternate low and high collision energy (CE) scans. Low CE was 5 V and elevated CE was ramped from 15 to 40 V. Ion mobility was implemented prior to fragmentation using a wave height of 650 m/s and wave velocity of 40V. The lockmass Glu[1]-Fibrinopeptide B ((M+2H)+2, *m/z* = 785.8426) was infused at a concentration of 100 fmol/µL at a flow rate of 250 nL/min and acquired every 60 s.

3. Database searches. Raw data were processed using a custom package (Regression tester) based upon executable files from ProteinLynx Global Server 3.0 (Waters). The optimal setting for peak detection across the dataset was determined using Threshold inspector (Waters) and these thresholds were applied: low energy = 100 counts; high energy = 30. Database searches were performed using regression tester and searched against the Uniprot human reference database (24/07/2017; 71,599 entries) with added sequence information for the internal standard Enolase. A maximum of two

missed cleavages was allowed for tryptic digestion with a fixed modification for carboxyamidomethylation of cysteine and a variable modification for the oxidation of methionine. Precursor and product ion mass tolerances were calculated automatically during data processing and the false discovery rate (FDR) was set at 4% and then filtered to 1% FDR. Quantity was estimated in absolute amounts (fmol) using the Top 3 method[1]. The ion accounting output files were compiled and summary information was generated from search log files using custom Python scripts. Information contained in ion accounting files was collated into a single .csv document using a custom Python script.

1. Silva JC, Gorenstein MV, Li GZ, Vissers JP, Geromanos SJ. Absolute quantification of proteins by LCMSE: a virtue of parallel MS acquisition. *Molecular & cellular proteomics : MCP* 2006, **5**(1)**:** 144-156.

Supplementary Figure Legends

Figure S1 Activation of EGFR signalling induces EMT in alveolar epithelial cells. (**a**) Differential gene expression and pathway analysis in IPF and normal lung tissue. Microarray data (GSE24206) was analysed using LIMMA software and differentially expressed genes determined using a Q value cut-off of 0.02. Gene network analysis using the Consensus Pathways Database was used to identify the pathways containing the differentially expressed genes. (**b**) Protein expression of E-cadherin, ZEB1 and phospho-ERK (p-ERK) in $ATII^{ER:KRASV12}$ treated with 100 ng/ml EGF or TGFα for 24 hrs. β-tubulin was used as a loading control. Scores under the bands are relative levels when compared with control (1.0).

Figure S2 Activation of RAS signalling induces EMT in alveolar epithelial cells. Representative phase contrast images of $ATII^{ER:KRASV12}$ cells cultured in Matrigel in the absence or presence of 250 nM 4-OHT for 48 hrs. Scale bars: 50 μm.

Figure S3 Activation of the RAS pathway drives EMT via ERK-ZEB1 in ATII cells. (**a**) Representative phase contrast images of $ATII^{ER:KRASV12}$ cells treated with 250 nM 4-OHT in absence or presence of inhibitors AKT VIII (10 μM) or U0126 (10 μM) for 24 hrs. DMSO was used as a vehicle control. 20 X magnification. (**b**) Representative phase contrast images of $ATII^{ER:KRASV12}$ cells transfected with the indicated siRNA followed by treatment of 250 nM 4-OHT for 24 hrs. 20 X magnification.

Figure S4 ATII cells undergoing RAS-induced EMT induce fibroblasts activation via paracrine signalling. (**a**) Fold change in mRNA levels of *COL1A1*, *COL3A1, FN1* and *ACTA2* in $ATII^{ER:KRASV12}$ or MRC5 cells with indicated treatments. β-actin-normalised mRNA levels in control ATII cells were used to set the baseline value at unity. Data

are mean ± s.d. $n = 3$ samples per group. (**b**) Fold change in mRNA levels of *COL1A1, FN1* and *ACTA2* in primary human lung fibroblasts from IPF (IPFFs) with indicated treatments. β-actin-normalised mRNA levels in control cells were used to set the baseline value at unity. Data are mean ± s.d. $n = 3$ samples per group. * $P < 0.05$. ** $P < 0.01$. *** $P < 0.001$.

Figure S5 ZEB1 is a key regulator of the paracrine signalling between ATII cells and fibroblasts. Fold change in mRNA levels of *COL1A1*, *COL3A1, FN1* and *ACTA2* in MRC5 cells with indicated treatments. β-actin-normalised mRNA levels in control cells were used to set the baseline value at unity. Data are mean ± s.d. $n = 3$ samples per group.

Figure S6 ZEB1 regulates the expression of tissue plasminogen activator (tPA), which acts as a paracrine regulator of TGFβ-induced fibroblast activation. (**a**) Diagram showing the *PLAT* promoter (-689 to -1 upstream of TSS, transcription start site) for cloning into the pGL3 basic construct. The amplified *PLAT* promoter region (-547 to -345) for ChIP, containing a ZEB1 binding site (5'-CANNTG-3') at -419 (highlighted in red, 5'-CAGGTG-3'), is underlined. (**b**) ChIP assays showing the ability of ZEB1 to bind the *PLAT* promoter in $ATII^{ER:KRASV12}$ cells in response to the indicated treatments. qPCR of fragments of the *PLAT* promoter immunoprecipitated in ChIP assays from $ATII^{ER:KRASV12}$ cells with an antibody against ZEB1 and control IgG. Amplified *PLAT* promoter region (-547~-345) contains a ZEB1 binding site at −419. (**c**) Protein expression of α-SMA and phospho-Smad2 (p-Smad2) in MRC5 lung fibroblasts treated with 10^{-8} M recombinant tPA in the presence or absence of 5 ng/ml TGFβ for 48 hrs. β-tubulin was used as a loading control. (**d**) Fold change in the mRNA level of *PLAT* (tPA) in $ATII^{ER:KRASV12}$ cells with indicated treatments. β- actin-normalised

mRNA levels in control cells were used to set the baseline value at unity. Data are mean ± s.d. $n = 3$ samples per group. *** $P < 0.001$.

Figure S7 Damaged ATII cells are a potential source of TGFβ. (**a**) Expression (*y*-axis) of *TGFB1*, *TGFB2* and *TGFB3* in IPF compared with control lung epithelial cells is shown from an online LGEA web portal (https://research.cchmc.org/pbge/lunggens/mainportal.html). (**b**) Expression (*y*-axis) of *SNAI1*, *SNAI2*, *SNAI3*, *TWIST1* and *TWIST2* in IPF *vs.* control epithelial cells is shown from an online LGEA web portal (https://research.cchmc.org/pbge/lunggens/mainportal.html). (**c**) Fold change in mRNA levels of *TGFB1* and *TGFB2* in $ATII^{ER:KRASV12}$ cells with indicated treatments. β-actin-normalised mRNA levels in control cells were used to set the baseline value at unity. Monolayers of $ATII^{ER:KRASV12}$ cells were scrape wounded with 8 or 12 scratches and allowed to repair for 24 hrs. Data are mean ± s.d. $n = 3$ samples per group. ** $P < 0.01$. *** $P < 0.001$.

Supplementary Table S1

List of proteins/genes that are up-regulated in the CM from 4-OHT-treated $ATII^{ER:KRASV12}$ cells identified by quantitative proteomic analysis and in IPF epithelial cells using an online LGEA web portal (https://research.cchmc.org/pbge/lunggens/mainportal.html).
