# Supplementary material for: Paracrine signalling during ZEB1-mediated epithelial–mesenchymal transition augments local myofibroblast differentiation in lung fibrosis
Source: Cell Death Differ. 2018 Jul 26;26(5):943–57. doi: 10.1038/s41418-018-0175-7 (PMC6252080; doi:10.1038/s41418-018-0175-7)

Supple Figure S1

a

P value	Q value	Pathway	Source
**6.76 x 10^{-8}**	**2.13 x 10^{-4}**	**EGFR1**	**NetPath**
1.05 x 10^{-6}	1.62 x 10^{-3}	FoxO signaling pathway - Homo sapiens	KEGG
1.54 x 10^{-6}	1.62 x 10^{-3}	Amoebiasis - Homo sapiens	KEGG
3.11 x 10^{-6}	2.45 x 10^{-3}	Interleukin-6 signaling	Reactome
6.73 x 10^{-6}	3.94 x 10^{-3}	Hemostasis	Reactome
7.51 x 10^{-6}	3.94 x 10^{-3}	Leptin	NetPath
1.93 x 10^{-5}	8.69 x 10^{-3}	Extracellular matrix organization	Reactome
2.41 x 10^{-5}	9.50 x 10^{-3}	p53 signaling pathway - Homo sapiens	KEGG
4.55 x 10^{-5}	1.57 x 10^{-2}	Leptin signaling pathway	Wikipathways
**5.00 x 10^{-5}**	**1.57 x 10^{-2}**	**MAPK1 (ERK2) activation**	**Reactome**
6.00 x 10^{-5}	1.72 x 10^{-2}	Thyroid hormone signaling pathway - Homo sapiens	KEGG
7.23 x 10^{-5}	1.87 x 10^{-2}	ECM-receptor interaction - Homo sapiens	KEGG
9.32 x 10^{-5}	1.87 x 10^{-2}	Platelet activation - Homo sapiens	KEGG
9.60 x 10^{-5}	1.87 x 10^{-2}	Retinoblastoma (RB) in Cancer	Wikipathways
1.81 x 10^{-4}	1.87 x 10^{-2}	Alpha6Beta4Integrin	NetPath
1.81 x 10^{-4}	1.87 x 10^{-2}	Ectoderm Differentiation	Wikipathways
**1.83 x 10^{-4}**	**1.87 x 10^{-2}**	**EGF-EGFR Signaling Pathway**	**Wikipathways**
2.25 x 10^{-4}	1.87 x 10^{-2}	Toxoplasmosis	Wikipathways
2.49 x 10^{-4}	1.87 x 10^{-2}	Polycystic Kidney Disease Pathway	Wikipathways

b

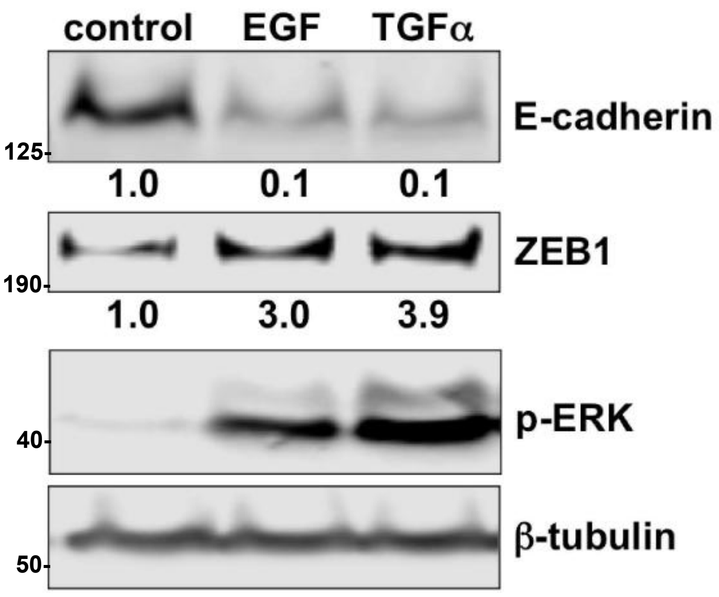

Supplement: Supplementary file 2 — Supplementary Fig. S1 [file 41418_2018_175_MOESM2_ESM.pdf]
