# Supplementary figures and images for: Paracrine signalling during ZEB1-mediated epithelial–mesenchymal transition augments local myofibroblast differentiation in lung fibrosis
Source: Cell Death Differ. 2018 Jul 26;26(5):943–57. doi: 10.1038/s41418-018-0175-7 (PMC6252080; doi:10.1038/s41418-018-0175-7)

## Supple Figure S2

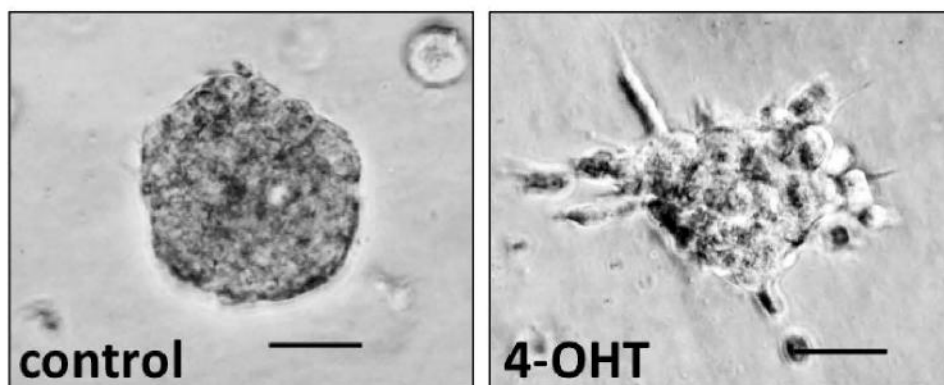

Supplement: Supplementary file 3 — Supplementary Fig. S2 [file 41418_2018_175_MOESM3_ESM.pdf]

## Supple Figure S3

**a**

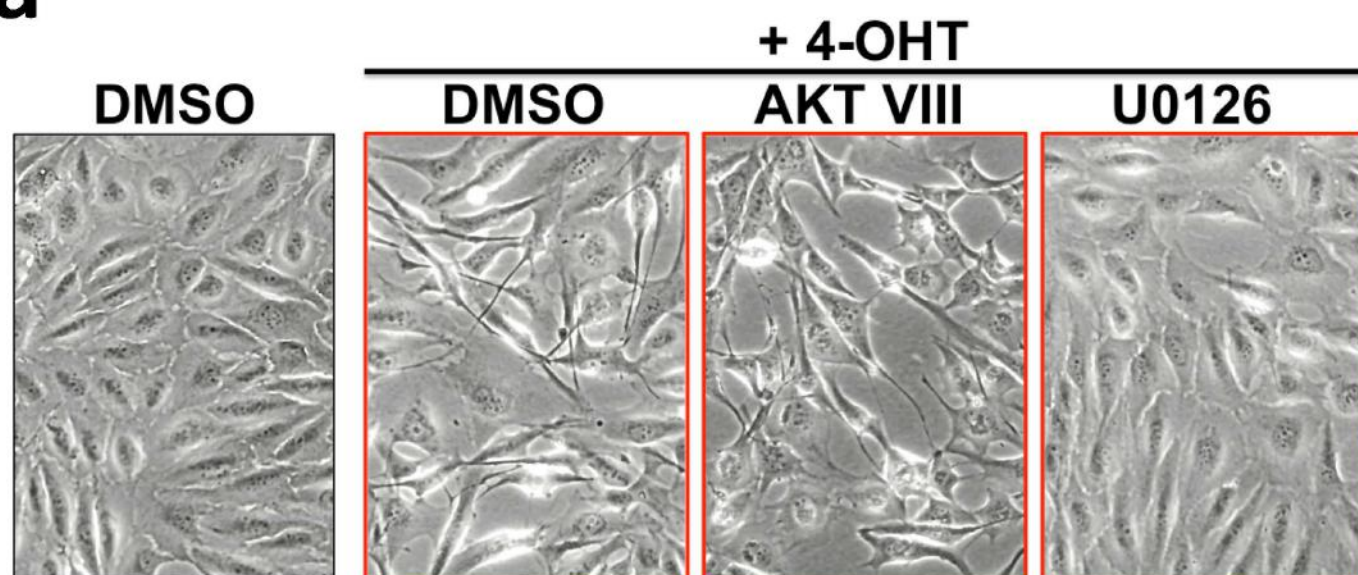

**b**

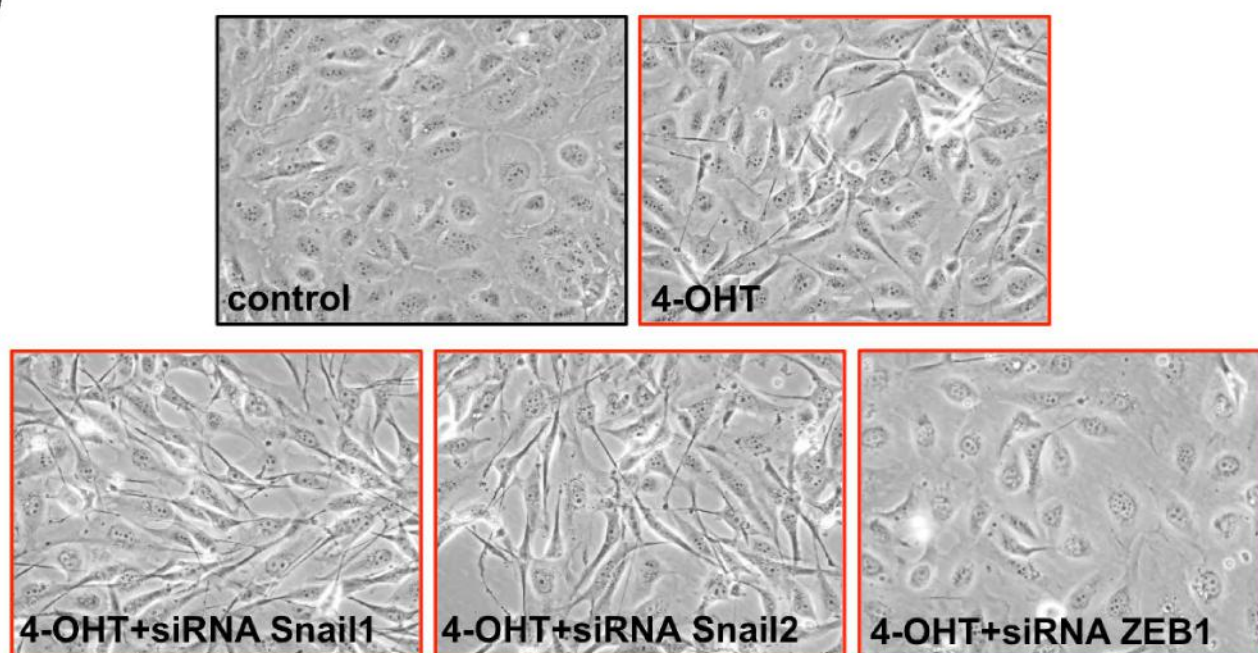

Supplement: Supplementary file 4 — Supplementary Fig. S3 [file 41418_2018_175_MOESM4_ESM.pdf]

# Supple Figure S4

**a**

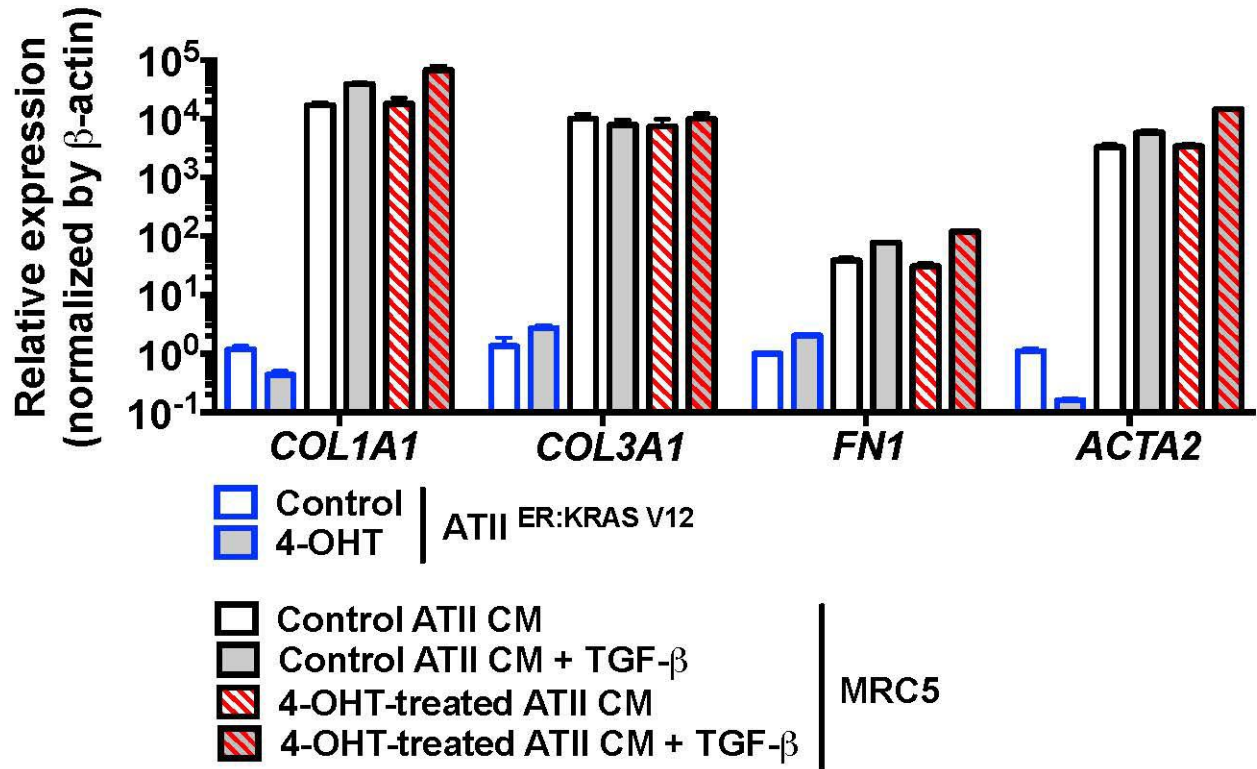

**b**

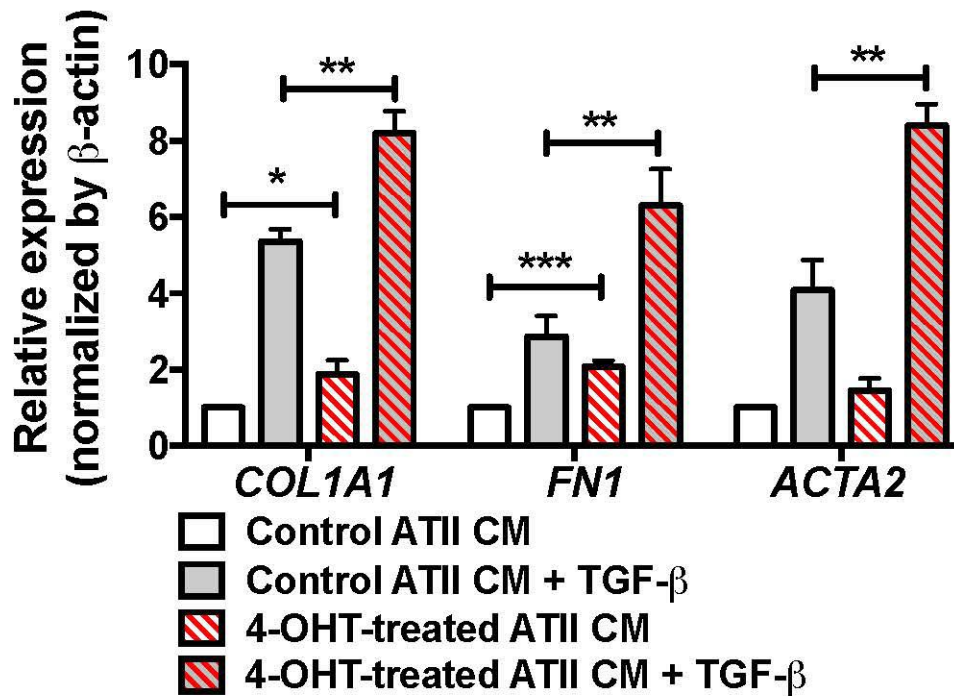

Supplement: Supplementary file 5 — Supplementary Fig. S4 [file 41418_2018_175_MOESM5_ESM.pdf]

## Supple Figure S5

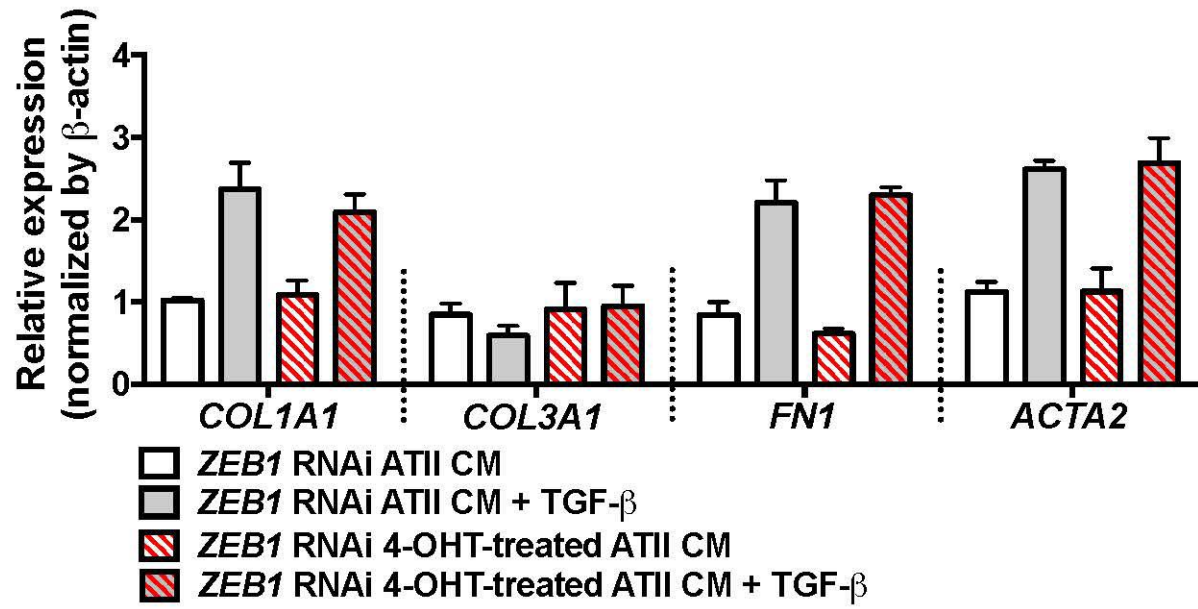

Supplement: Supplementary file 6 — Supplementary Fig. S5 [file 41418_2018_175_MOESM6_ESM.pdf]

## Supple Figure S7

**a**

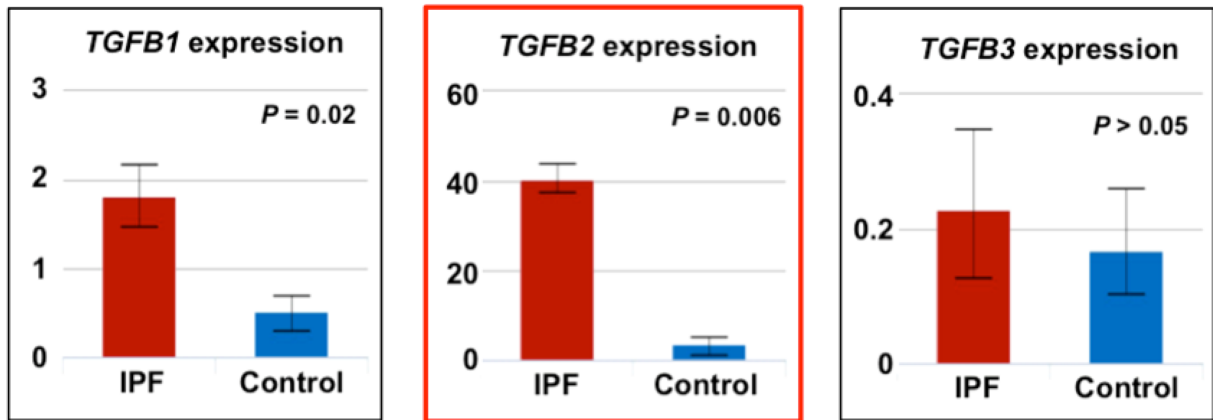

**b**

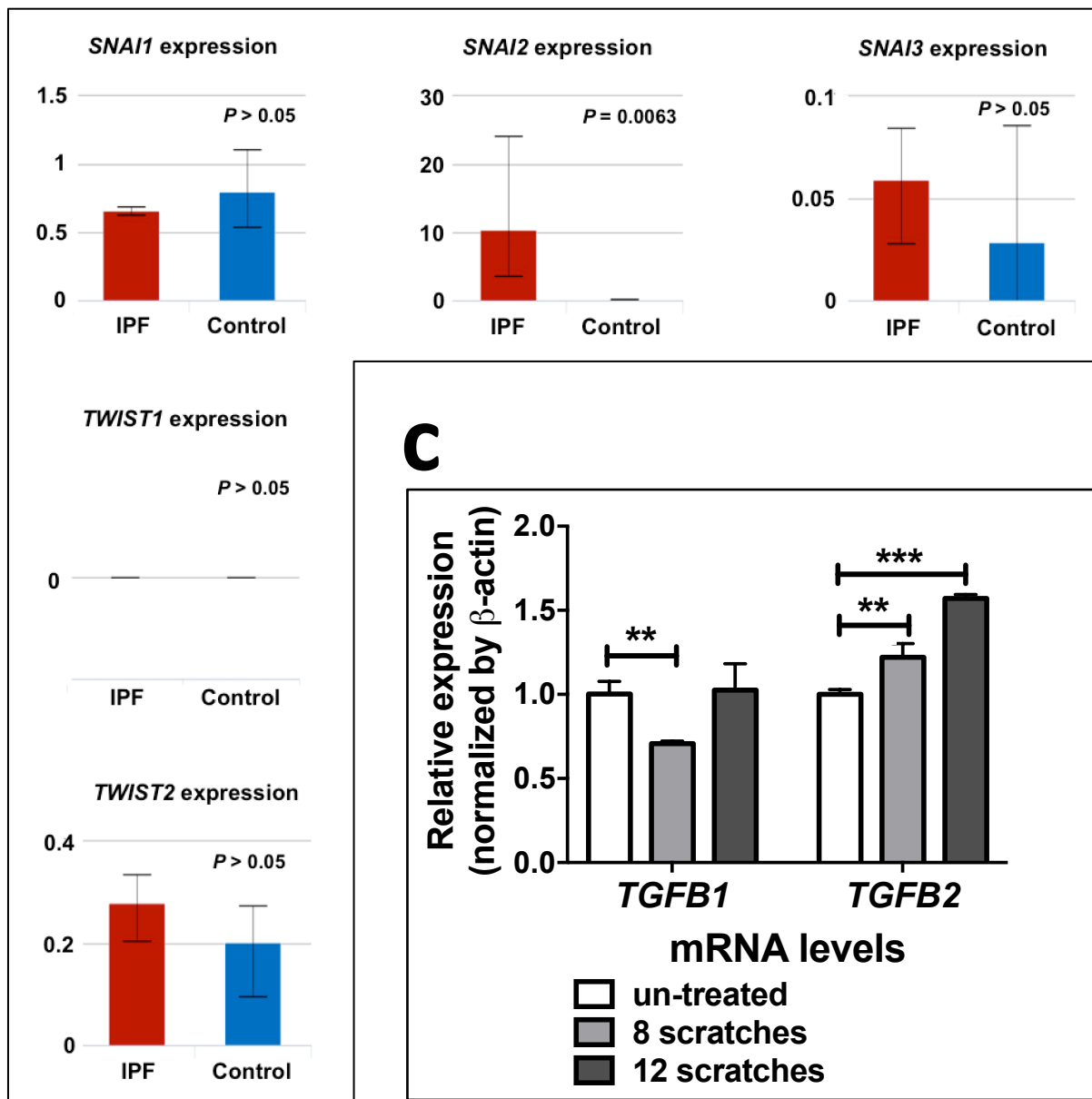

**c**

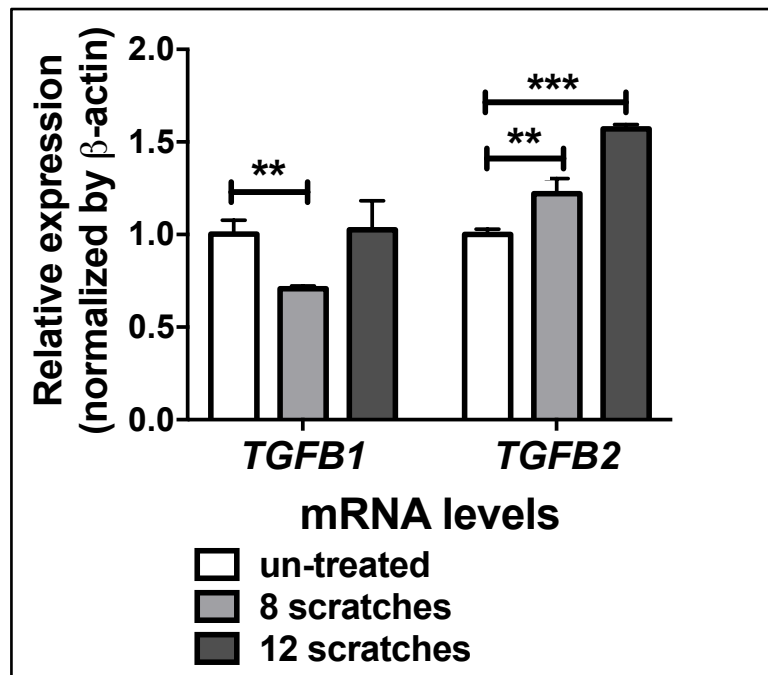

Supplement: Supplementary file 8 — Supplementary Fig. S7 [file 41418_2018_175_MOESM8_ESM.pdf]
