# Supplementary material for: Paracrine signalling during ZEB1-mediated epithelial–mesenchymal transition augments local myofibroblast differentiation in lung fibrosis
Source: Cell Death Differ. 2018 Jul 26;26(5):943–57. doi: 10.1038/s41418-018-0175-7 (PMC6252080; doi:10.1038/s41418-018-0175-7)

Supple Figure S6

a

***PLAT* promoter for cloning (-689 to -1 upstream of TSS/bp)**
[ZEB1 binding site E box (5-CANNTG-3)]

5'TCTATTTCCAGGGGCATTTTACAAGCAAATACTGAAAGGCTTCGGTGGGCTTAAGGGCTGATGGCTTTG
ATCGAATTTCAGGCATGTTGGCCCCAAGGCCCTGTGTATATTCCCTGGGCCCACTCAAGGGGATGCTGG
AGCCGGAAAGTCCCCGGAGGCCACCTACTGCAGCCCTGCACTTTACAAAGAAGAGAAAGATTCTCCCTA
AAATTACAGAACAGGGCCAAAGATGCCTACCGGAGCAAACCCCCATGGGGGCACCTCCTACCGCAGGTG
AGCCCAAGGCTGGTCCTGCCTTCTCAGTGGCTACCCCCCTGAGCTCCCGCCACCACACAAAGTGTTCCA
ATCCTTGTGCATCCTCCAGTCCTTTTAACCTCTCATGTCCTGAGAGGCCAGAGCTACAGCCACAGATTCC
AGAAGACACCCCACTCCCAGCCCCAACCTGCTGCCTTTAGAATTATAAACACTTCTTGTCATCACAGGGT
CCTGAAAGTCCCTTTTAAGCCTGGGACACTAGGACTCTAAAGGAAGATGATTCTTAAGGTCCCATCCCAC
TTCCAAATTCCTGCGATTCAATGACATCACGGCTGTGAATAATCAGCCTGGCCCGAAGCCAGGATGGGCT
GTGCTGCTTCCACCGTGAACTTCCTCCCCCTGCTTTATAAAAACAGGCCTGCCTCAGCTCCCTC3'

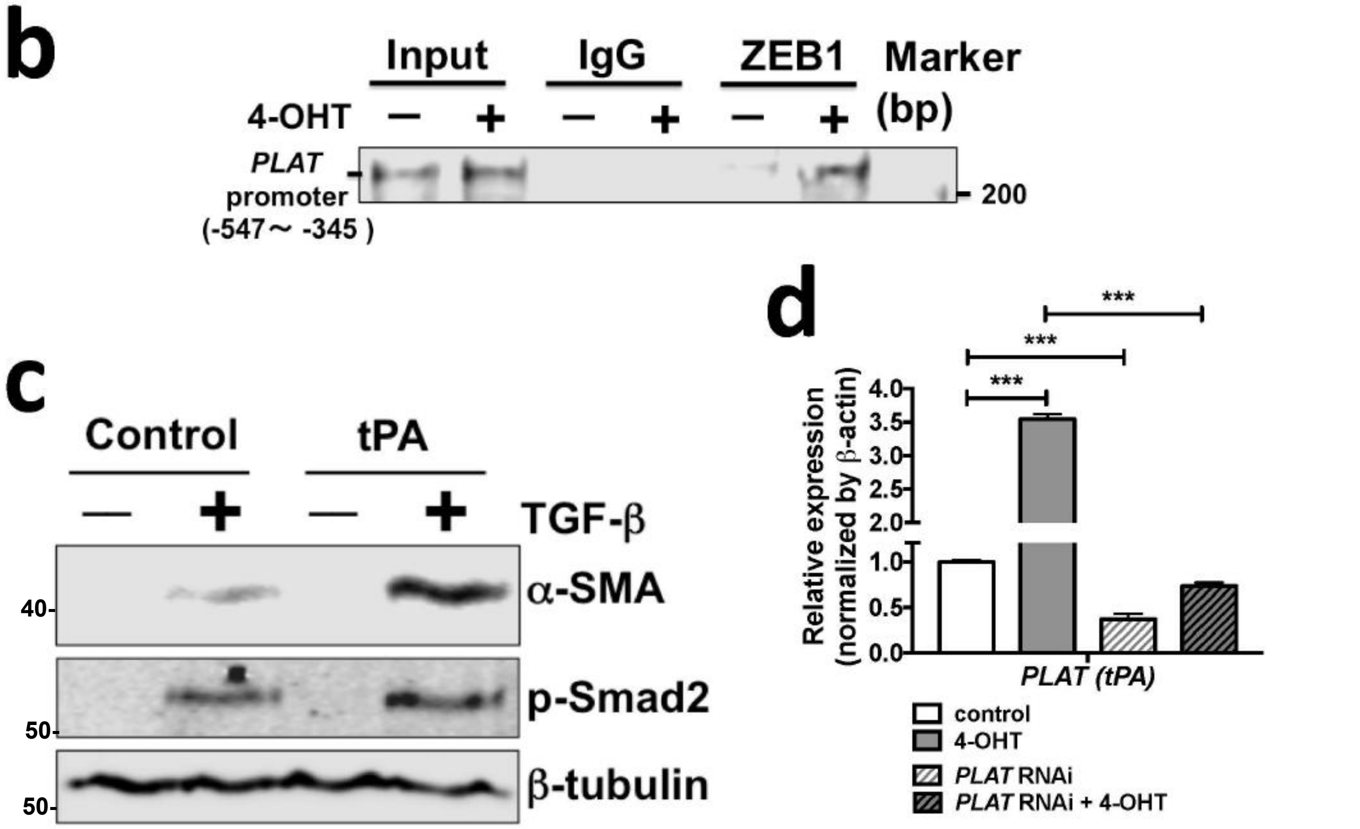

Supplement: Supplementary file 7 — Supplementary Fig. S6 [file 41418_2018_175_MOESM7_ESM.pdf]
