# Supplementary material for: Paracrine signalling during ZEB1-mediated epithelial–mesenchymal transition augments local myofibroblast differentiation in lung fibrosis
Source: Cell Death Differ. 2018 Jul 26;26(5):943–57. doi: 10.1038/s41418-018-0175-7 (PMC6252080; doi:10.1038/s41418-018-0175-7)
Supplement: Supplementary file 9 — Supplementary Table S1 [file 41418_2018_175_MOESM9_ESM.pdf]

Supplementary Table S1

Secretome analysis																LGEA analysis	
protein.Entry	protein.Accession	protein.Description	Control 1	Control 2	Control 3	Mean Control	4OHT_1	4OHT_2	4OHT_3	Mean 4OHT	Present_NumFiles	4OHT/Control	*t* test			*P* value	fold change
sp	P00750	TPA_HUMAN Tissue-type plasminogen activator OS=Homo sapiens GN=PLAT PE=1 SV=1	0.03805945	0.01625878	1.42580102	0.493373083	5.43590936	3.76467321	3.91557126	4.37205128	4	8.86155209	0.0054235			0.0029	62.186
sp	O43665	RGS10_HUMAN Regulator of G-protein signaling 10 OS=Homo sapiens GN=RGS10 PE=1 SV=2	0.03805945	0.01625878	0.07417637	0.042831532	0.99216271	0.780106	0.60354859	0.7919391	3	18.48962817	0.0027397			0.0173	31.993
sp	Q9UBP4	DKK3_HUMAN Dickkopf-related protein 3 OS=Homo sapiens GN=DKK3 PE=1 SV=2	5.93056295	5.83114799	4.56300733	5.441572754	10.0672589	9.12225748	6.76727499	8.65226378	6	1.590029974	0.0405159			0.0016	22.862
sp	P09382	LEG1_HUMAN Galectin-1 OS=Homo sapiens GN=LGALS1 PE=1 SV=2	26.24369	24.4859987	25.7932936	25.50766077	32.8702499	34.1803729	33.0724352	33.3743527	6	1.30840507	0.0002942			0.0078	21.273
sp	P49006	MRP_HUMAN MARCKS-related protein OS=Homo sapiens GN=MARCKSL1 PE=1 SV=2	0.03805945	0.01625878	0.07417637	0.042831532	1.22915429	0.81883881	1.03388924	1.02729411	3	23.98452876	0.0011912			0.0448	8.948
sp	Q9BUF5	TBB6_HUMAN Tubulin beta-6 chain OS=Homo sapiens GN=TUBB6 PE=1 SV=1	1.59108512	1.6288924	1.70757031	1.642515942	2.42474405	1.99892431	1.96350613	2.12905816	6	1.296217653	0.0329479			0.0085	7.791
sp	Q01813	PFKAP_HUMAN ATP-dependent 6-phosphofructokinase_ platelet type OS=Homo sapiens GN=PFKP PE=1 SV=2	1.41330745	1.34007068	0.99123851	1.248205545	2.99812274	2.50893872	2.45329649	2.65345265	6	2.12581386	0.0029099			0.0103	4.928
sp	O00154	BACH_HUMAN Cytosolic acyl coenzyme A thioester hydrolase OS=Homo sapiens GN=ACOT7 PE=1 SV=3	5.52472907	4.71961366	5.08920541	5.111182716	7.68256975	8.53283108	7.77277898	7.99605994	6	1.564424592	0.0012627			0.0157	4.724
sp	Q16643	DREB_HUMAN Drebrin OS=Homo sapiens GN=DBN1 PE=1 SV=4	1.28240299	1.27421348	0.07417637	0.876930945	2.36175646	2.03966836	1.9269384	2.10945441	5	2.405496598	0.0432139			0.0027	4.643
sp	P08727	K1C19_HUMAN Keratin_ type I cytoskeletal 19 OS=Homo sapiens GN=KRT19 PE=1 SV=4	16.5180996	12.9025304	16.2798462	15.23349207	23.4078944	23.8995049	26.3436831	24.5503608	6	1.611604266	0.003245			0.0206	4.214
sp	O95865	DDAH2_HUMAN N(G)_N(G)-dimethylarginine dimethylaminohydrolase 2 OS=Homo sapiens GN=DDAH2 PE=1 SV=1	0.03805945	0.59171003	0.07417637	0.234648615	1.22556913	3.90442274	4.6763973	3.26879639	4	13.93060167	0.0459206			0.0237	3.299
sp	P08729	K2C7_HUMAN Keratin_ type II cytoskeletal 7 OS=Homo sapiens GN=KRT7 PE=1 SV=5	4.32725875	4.56625134	3.79641725	4.229975778	7.48748748	8.05830553	8.50840587	8.01806629	6	1.8955348	0.0005285			0.0013	3.216
sp	Q9NVA2	SEP11_HUMAN Septin-11 OS=Homo sapiens GN=SEPT11 PE=1 SV=3	1.57415868	1.13117274	0.97297195	1.226101122	2.2307126	3.16447689	2.8112982	2.7354959	6	2.231052436	0.0098376			0.004	2.985
sp	Q16555	DPYL2_HUMAN Dihydropyrimidinase-related protein 2 OS=Homo sapiens GN=DPYSL2 PE=1 SV=1	2.68078712	2.34254329	2.10650798	2.376612799	3.84755891	3.69629067	4.17248673	3.90544543	6	1.643282169	0.0021756			0.009	2.707
sp	Q96HC4	PDLI5_HUMAN PDZ and LIM domain protein 5 OS=Homo sapiens GN=PDLIM5 PE=1 SV=5	0.03805945	0.01625878	0.07417637	0.042831532	1.42312393	1.51615911	1.08566335	1.34164879	3	31.32385725	0.000596			0.0108	2.637
sp	P14618	KPYM_HUMAN Pyruvate kinase PKM OS=Homo sapiens GN=PKM PE=1 SV=4	34.1364177	34.2956157	41.1210583	36.51769721	44.7813329	41.5010682	46.4058265	44.2294092	6	1.211177391	0.046935			0.018	2.134
sp	Q15942	ZYX_HUMAN Zyxin OS=Homo sapiens GN=ZYX PE=1 SV=1	4.62041663	5.84959897	4.55543831	5.008484634	7.11327073	6.31643212	7.42846302	6.95272195	6	1.388188736	0.0221407			0.0048	2.119
sp	P33316	DUT_HUMAN Deoxyuridine 5'-triphosphate nucleotidohydrolase_ mitochondrial OS=Homo sapiens GN=DUT PE=1 SV=4	0.66413732	0.01625878	1.08337868	0.587924926	3.09795096	1.77269097	2.29507769	2.38857321	5	4.062718048	0.0219907			0.0262	2.049
sp	Q9H299	SH3L3_HUMAN SH3 domain-binding glutamic acid-rich-like protein 3 OS=Homo sapiens GN=SH3BGRL3 PE=1 SV=1	10.6936023	11.6126062	10.6132761	10.97316155	13.5005461	13.5527846	12.8667001	13.306677	6	1.212656616	0.0038884			0.0329	1.971
sp	Q9HA64	KT3K_HUMAN Ketosamine-3-kinase OS=Homo sapiens GN=FN3KRP PE=1 SV=2	0.03805945	0.01625878	0.07417637	0.042831532	0.8801573	1.3146447	1.45076137	1.21518779	3	28.3713361	0.0024689			0.011	1.845
sp	P35237	SPB6_HUMAN Serpin B6 OS=Homo sapiens GN=SERPINB6 PE=1 SV=3	1.84898793	2.04193852	1.8771163	1.922680918	2.79704459	2.88861106	3.64011849	3.10859138	6	1.616800455	0.0123326			0.0399	1.781
sp	P67936	TPM4_HUMAN Tropomyosin alpha-4 chain OS=Homo sapiens GN=TPM4 PE=1 SV=3	6.7261055	6.07247215	7.76056393	6.853047193	9.1039629	8.09742761	8.41492282	8.53877111	6	1.245981659	0.0425782			0.0447	1.718
sp	P15311	EZRI_HUMAN Ezrin OS=Homo sapiens GN=EZR PE=1 SV=4	24.1979949	22.4183019	21.3273715	22.64788942	26.6794785	30.7285466	30.7930708	29.4003653	6	1.298150337	0.0133946			0.0204	1.679
sp	O14907	TX1B3_HUMAN Tax1-binding protein 3 OS=Homo sapiens GN=TAX1BP3 PE=1 SV=2	0.03805945	0.01625878	0.07417637	0.042831532	1.88357001	4.36947595	4.53555723	3.59620106	3	83.96153243	0.0143496			0.0023	1.585
sp	O43707	ACTN4_HUMAN Alpha-actinin-4 OS=Homo sapiens GN=ACTN4 PE=1 SV=2	24.6135439	25.0097507	25.5202034	25.04783267	32.0447354	30.9006059	34.5628056	32.5027156	6	1.297625869	0.0025856			0.0246	1.507
